# FSF-RTDETR: a frequency–spatial feature fusion network for eggplant fruit disease and pest detection in complex agricultural scenes

**DOI:** 10.3389/fpls.2026.1956953

**Published:** 2026-10-23

**Authors:** Hui Sun, Zhijun Zhang, Jun Liu, Delong Zhang, Minghan Feng

**Affiliations:** Weifang University of Science and Technology, Weifang, China

**Keywords:** eggplant disease and pest detection, frequency-domain enhancement, frequency-spatial fusion, mamba, RT-DETR

## Abstract

Detecting fruit diseases and pests in complex agricultural scenes remains challenging due to large target-scale variation, weak lesion features, leaf and branch occlusion, and severe background interference. To address these problems, this study focuses on eggplant fruit disease and pest detection and proposes FSF-RTDETR, a frequency–spatial fusion model. The proposed model builds a dual-branch frequency–spatial enhanced backbone to improve disease and pest feature representation from both frequency details and spatial structures. In the frequency branch, AFDC and MFFWBlock enhance high-frequency responses such as fruit borer edges, rot lesion textures, and thrips bite marks through attention-guided frequency-domain convolution and multiscale wavelet feature modeling. This design improves the perception of small targets with weak boundaries and fine textures. In the spatial branch, VisionClueMerge and G6MambaLayer preserve local spatial cues and introduce six directional scanning paths covering horizontal, vertical, and diagonal interactions. This helps the model better describe occluded targets, complex backgrounds, and irregular lesion distributions. Furthermore, EGMuF adaptively integrates edge-texture information from the frequency branch and structural semantic information from the spatial branch through channel, spatial, and pixel-level gated attention. Experimental results show that FSF-RTDETR achieves Precision, Recall, mAP_50_, and mAP_50:95_ of 81.5%, 71.3%, 78.8%, and 50.7% on the Eggplant dataset, respectively, outperforming the RT-DETR-R18 baseline by 1.7, 2.0, 4.2, and 4.7 percentage points. The model achieves an inference speed of 104.07 FPS. Results on the PlantDoc Dataset further verify the effectiveness and generalization ability of the proposed method in plant leaf disease detection scenarios.

## 1 Introduction

Crop diseases and pests are among the major factors affecting agricultural production stability and the quality of agricultural products. Timely and accurate identification of disease and pest regions is important for suppressing disease spread, reducing pesticide use, and ensuring crop yield (Kumar and Raghavendra, 2019). In recent years, the rapid development of artificial intelligence and deep learning has promoted the wide application of machine vision in precision agriculture. Vision-based disease and pest detection methods can work with agricultural robots, UAV spraying systems, and intelligent monitoring equipment to achieve automatic localization and precise control of affected regions. These methods provide important technical support for green agriculture and smart agriculture (Martinelli et al., 2015; Meshram et al., 2022). Convolutional neural networks (CNNs) have inductive biases such as local connectivity, weight sharing, and translation equivariance. These properties help CNNs extract visual features such as edges, textures, and local shapes. Therefore, CNNs have been widely used in crop disease and pest recognition and detection tasks (LeCun et al., 2015; Liu et al., 2020; Aslan et al., 2026). For example, Ashok et al. (2020) used deep learning methods to detect tomato leaf diseases. Khanramaki et al. (2021) adopted an ensemble of deep learning models for citrus pest classification. Qi et al. (2022) improved You Only Look Once (YOLO)v5 for tomato disease detection. Atila et al. (2021) used EfficientNet for plant leaf disease classification. Bari et al. (2021) employed a Faster R-CNN model to identify rice leaf diseases. Nawaz et al. (2024) applied deep learning methods to recognize coffee leaf diseases and pests. These studies show that deep learning methods can improve the automation level of crop disease and pest recognition and reduce the cost of manual field inspection.

Eggplant (*Solanum melongena* L.) is an important solanaceous cash crop with high nutritional and horticultural value (Shi et al., 2018). However, under natural growing conditions, eggplant fruits are easily affected by fruit borers, rot lesions, and thrips bite marks, which reduce fruit quality and cause yield losses. Existing studies on eggplant disease and pest detection have mostly focused on improving YOLO-based models (Redmon et al., 2016). For example, Sun et al. (2025a) proposed an eggplant disease detection model based on YOLOv11. Sun et al. (2026) developed DFSNet, a YOLOv11-based model for eggplant fruit disease and pest detection. Huang et al. (2024) proposed YOLOv8-E for eggplant disease and pest detection. Xie et al. (2024) designed YOLOv5s-BiPCNeXt for eggplant leaf disease detection. Liu and Wang (2025) proposed EggplantDet based on YOLOv8. These methods offer fast detection and relatively low deployment costs. However, they still face challenges in complex field environments, such as weak lesion features, large target-scale variation, severe leaf and branch occlusion, and strong background interference. In addition, YOLO-based detectors usually rely on dense prediction filtering and nonmaximum suppression (NMS) postprocessing. In scenes with small targets, occluded targets, or many adjacent targets, NMS may suppress some low-confidence but real disease and pest targets, which affects detection stability. In contrast, Transformer-based RT-DETR removes NMS through an end-to-end detection framework. It also combines the local feature extraction ability of CNNs with the global modeling ability of Transformers, providing a new idea for object detection in complex agricultural scenes (Vaswani et al., 2017; Zhao et al., 2024; Dong et al., 2026; Han et al., 2026a). Existing studies (Huangfu et al., 2024; Liu et al., 2025; Sun et al., 2025b; Han et al., 2026b, 2026c; Huo et al., 2026) have also improved RT-DETR for eggplant disease and pest detection and achieved good detection performance. These studies have mainly explored spatial-domain feature enhancement, attention mechanisms, feature fusion, and lightweight architectural design. Although these improvements strengthen feature representation, the visual information used for detection is not limited to spatial structures. Images also contain complementary frequency components that describe variations at different spatial scales (Mallat, 1989). They still make insufficient use of frequency information in images. In fact, visual images contain not only spatial structural information but also rich frequency components (Mallat, 1999; Aslan et al., 2026). In eggplant fruit disease and pest images, fruit borer edges, rot lesion textures, and thrips bite marks often appear as fine-grained high-frequency responses, whereas fruit morphology, occlusion relationships, and contextual dependencies among fruits, leaves, and branches require effective spatial structure modeling. Recent studies (Sun et al., 2025c) have demonstrated that frequency-domain enhancement, wavelet transforms, and Fourier-based operations can improve object detection by introducing information that is complementary to conventional spatial-domain representations. Frequency-aware methods can strengthen high-frequency responses associated with object boundaries and fine textures, wavelet transforms can decompose features into multiple frequency components and facilitate multiscale representation, and Fourier-based operations can model global spectral characteristics and long-range feature dependencies. These approaches have shown that frequency information can provide useful discriminative cues beyond those extracted solely in the spatial domain. This is particularly important for agricultural images because discriminative disease and pest cues are jointly determined by fine-grained details, such as lesion boundaries and insect damage, and by their spatial relationships with fruits, leaves, branches, and surrounding regions. In contrast, occlusion relationships, fruit morphology, and background context depend more on spatial structure modeling. A single spatial-domain feature extraction strategy struggles to capture both high-frequency texture details of weak lesions and global context under complex backgrounds, which may lead to missed detections and false detections. Therefore, existing methods have not fully explored the complementary relationship between frequency-domain details and spatial contextual information. To address these problems, this study proposes Frequency–Spatial Fusion RT-DETR (FSF-RTDETR), a frequency–spatial feature fusion model for eggplant fruit disease and pest detection, with RT-DETR-R18 as the baseline. The main contributions of this study are as follows:

- This study proposes FSF-RTDETR for eggplant fruit disease and pest detection. The model builds a dual-branch frequency–spatial enhanced backbone to improve feature representation from both frequency responses and spatial structures in complex natural scenes.
- A frequency-domain enhancement branch is designed to strengthen fine-grained disease and pest features. This branch introduces Attention-guided Frequency-Domain Convolution (AFDC) and Multiscale Frequency Fusion Wavelet Block (MFFWBlock). It uses frequency-domain convolution and multiscale wavelet feature modeling to enhance high-frequency details, including fruit borer edges, rot lesion textures, and thrips bite marks. This improves the perception of small, weak-boundary, and fine-textured targets.
- A spatial enhancement branch is designed to improve contextual modeling in complex scenes. This branch combines VisionClueMerge and G6MambaLayer to preserve local spatial cues. It further introduces a six-direction selective scanning mechanism to capture horizontal, vertical, and diagonal spatial dependencies. This design improves detection performance under occlusion, small-lesion, and complex-background conditions.
- EGMuF, an enhanced frequency–spatial feature fusion module, is proposed. Through channel, spatial, and pixel-level gated attention, EGMuF adaptively fuses frequency-domain detail features and spatial semantic features. It can dynamically select effective information in different disease and pest scenes and achieve complementary representation of edge-texture details and spatial contextual information.
- Experiments are conducted on the public Eggplant dataset and PlantDoc dataset. The results verify the effectiveness and generalization ability of the proposed method in eggplant fruit disease and pest detection and complex plant disease detection scenarios.

## 2 Related work

### 2.1 Deep learning-based object detection and RT-DETR

With the development of deep learning, vision-based plant disease and pest recognition has gradually shifted from traditional hand-crafted feature methods to data-driven deep feature learning methods. Early studies mostly used convolutional neural networks to classify disease images of plant leaves or fruits, demonstrating the effectiveness of deep learning in automatic plant disease diagnosis (Mohanty et al., 2016; Ferentinos, 2018). However, classification models usually provide only image-level prediction results and cannot indicate the specific locations of disease and pest regions. Therefore, object detection models have been introduced into plant disease and pest recognition tasks to perform category identification and target localization simultaneously (Fuentes et al., 2017). Current deep learning-based object detection methods mainly include one-stage detectors, two-stage detectors, and Transformer-based end-to-end detectors. The YOLO series is a typical one-stage detection method. It has the advantages of fast inference speed and low deployment cost, and has been widely used in agricultural object detection tasks (Redmon et al., 2016). Two-stage detectors usually first generate candidate regions and then perform classification and bounding box regression. They often show strong localization ability, but their inference process is relatively complex. Unlike these methods, DETR formulates object detection as a set prediction problem. It uses Hungarian bipartite matching to establish one-to-one assignments between predicted boxes and ground-truth boxes, enabling end-to-end detection and removing anchor design and NMS postprocessing (Carion et al., 2020). Based on DETR, RT-DETR further designs an efficient hybrid encoder and a query selection strategy. These designs reduce encoding computational cost and improve query quality, thereby improving real-time detection performance while maintaining the advantages of end-to-end detection (Zhao et al., 2024). Therefore, RT-DETR combines CNN-based local feature extraction, Transformer-based global modeling, and end-to-end prediction, making it suitable as a baseline framework for object detection tasks in complex agricultural scenes. In this study, we further improve the RT-DETR framework to enhance the model’s ability to represent fine-grained textures, spatial dependencies, and multisource feature fusion.

### 2.2 Frequency-domain enhancement and multiscale wavelet modeling

In recent years, a number of excellent studies (Wang et al., 2023, 2024b, 2025a; Li et al., 2026; Liu et al., 2026) have emerged in the field of image analysis, providing important references for this work. Visual images contain not only spatial structural information but also rich frequency components. Mallat (1989) proposed that image features can be represented at different scales and in the frequency domain, which helps describe structural information and local details more fully. Traditional convolution mainly extracts local edge and texture features in the spatial domain, but it can be affected by background noise, illumination changes, and local texture similarity. In complex natural scenes, the subtle differences between small targets and background regions can be weakened during feature extraction (Yin et al., 2019; Singh et al., 2020). In contrast, frequency-domain features describe edge variations, fine-grained textures, and local abnormal information from the frequency perspective. Existing studies have shown that introducing wavelet decomposition or frequency-domain convolution into convolutional neural networks can enhance the modeling of different frequency subbands and improve texture representation, detail recovery, and global frequency response expression (Fujieda et al., 2018; Liu et al., 2018; Chi et al., 2020). Fast Fourier Convolution introduces frequency-domain transformation, allowing networks to model local spatial information and global frequency responses simultaneously, which helps enhance texture structures and high-frequency details (Chi et al., 2020). Wavelet modeling further provides an effective way for multiscale detail representation. Wavelet Convolutional Neural Networks introduce wavelet decomposition into convolutional networks to enhance feature representation in different frequency subbands (Fujieda et al., 2018). Multilevel Wavelet-CNN further shows that multilevel wavelet decomposition helps preserve image structural information and local details (Liu et al., 2018). Therefore, this study introduces FDConv and multiscale wavelet modeling into the frequency-domain enhancement branch to strengthen the edge-texture representation of small disease and pest targets, such as fruit borer edges, rot lesion textures, and thrips bite marks.

### 2.3 Spatial dependency modeling and gated feature fusion

Traditional convolution mainly relies on local receptive fields and has limited ability to model long-range spatial dependencies. Nonlocal Neural Networks enhance the capture of long-range dependency information by explicitly computing relationships between different positions (Wang et al., 2018). In recent years, Mamba has achieved efficient long-sequence modeling through a selective state space mechanism (Gu and Dao, 2023). VMamba further introduces state space modeling into vision tasks and enhances spatial dependency representation through two-dimensional selective scanning (Liu et al., 2024).VMamba usually performs feature propagation through multidirectional two-dimensional selective scanning, which can strengthen spatial interactions in horizontal and vertical directions. However, in natural agricultural scenes, fruits, lesions, leaves, branches, and backgrounds are often irregularly distributed. Lesion edges, branch orientations, and fruit contours also often show diagonal or curved patterns. Conventional scanning paths alone may not fully cover such irregular spatial relationships. Therefore, this study further introduces a six-direction selective scanning mechanism into the spatial enhancement branch to improve the model’s ability to represent occluded targets, small lesions, and complex backgrounds. In addition, different types of features contribute differently to disease and pest detection. Small lesions, pest bite marks, and rot edges rely more on high-frequency textures and local details, whereas heavily occluded targets or targets in complex backgrounds rely more on spatial semantics and contextual information. Therefore, adaptive feature fusion is important for improving detection robustness. SE Networks perform feature recalibration through channel attention (Hu et al., 2018). CBAM further combines channel attention and spatial attention, enabling the network to focus on both key channels and important spatial regions (Woo et al., 2018). These studies show that attention mechanisms can effectively enhance the network’s ability to select key features, and they also provide a reference for spatial dependency modeling and adaptive frequency–spatial fusion in this study.

## 3 Methods

### 3.1 Overview of FSF-RTDETR

The overall architecture of the model is shown in **Figure 1**. FSF-RTDETR mainly consists of three parts: a dual-branch frequency–spatial enhanced backbone, a multiscale feature interaction and fusion network, and a detection decoder head. First, the input image passes through the dual-branch frequency–spatial enhanced backbone for feature extraction. This backbone contains a frequency enhancement branch and a spatial enhancement branch. The frequency enhancement branch focuses on mining high-frequency textures, weak edges, and local abnormal responses in disease and pest regions. The spatial enhancement branch focuses on capturing spatial structural relationships and contextual information among fruits, lesions, leaves, branches, and backgrounds. The two branches are adaptively fused by the EGMuF module at multiple scale stages, producing multiscale features P3, P4, and P5. This process achieves complementary representations of frequency-domain detail information and spatial semantic information. The multiscale feature interaction and fusion network performs cross-scale semantic interaction and information fusion on P3, P4, and P5, and feeds the enhanced features into the detection decoder head. The detection decoder head selects high-quality queries through Intersection over Union (IoU)-aware Query Selection and generates the final detection results using decoder layers and a matching strategy.

**Figure 1:**
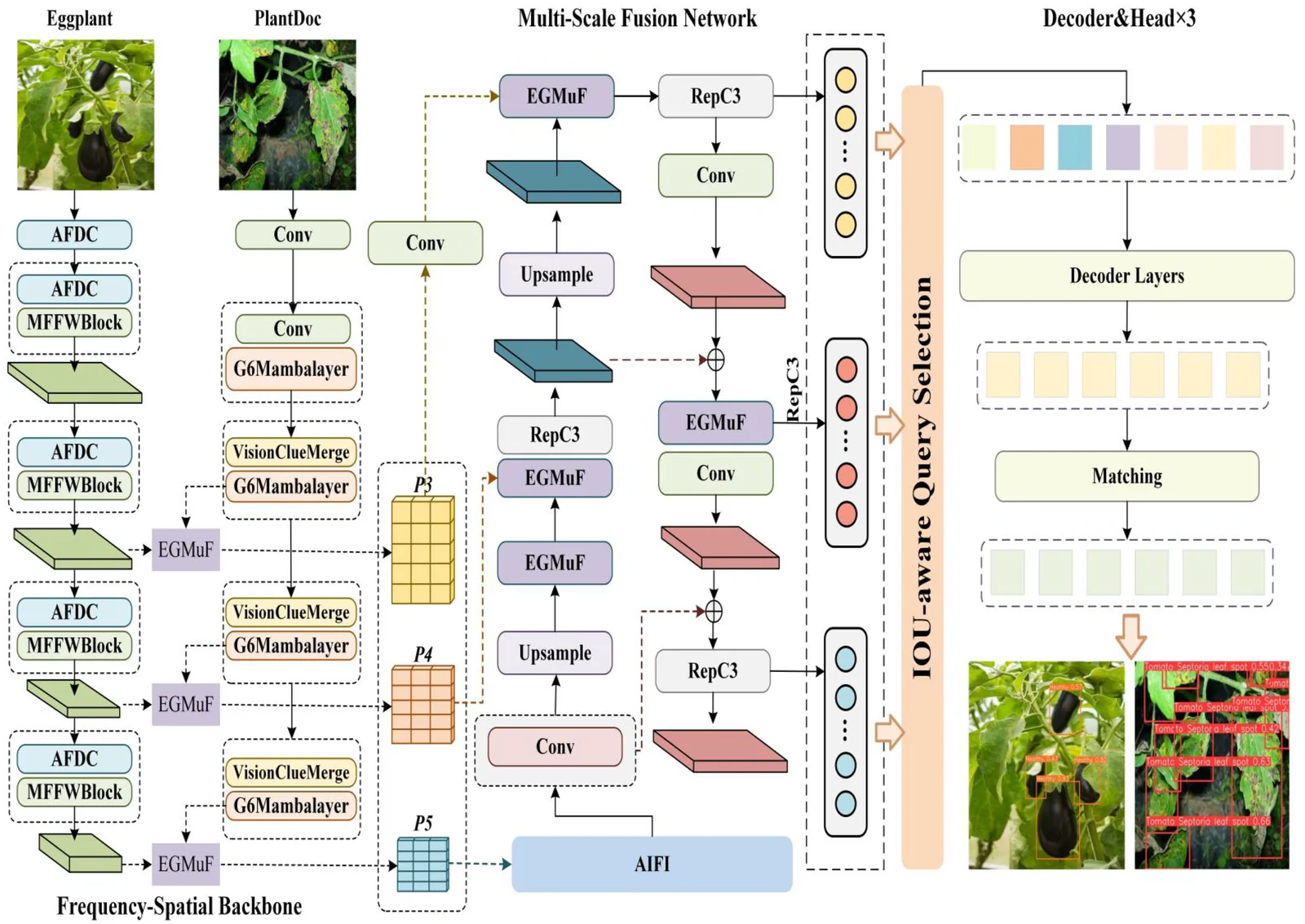
Overall architecture of FSF-RTDETR.

### 3.2 Frequency-domain enhancement branch

In images of eggplant fruit diseases and pests, fruit borer holes, thrips bite marks, and early rot lesions usually show small size, weak edges, and fine textures. During downsampling, these detailed features can be weakened, which may affect subsequent localization and recognition results. To improve the model’s perception of high-frequency details and local abnormal responses, this study builds a frequency-domain enhancement branch in the dual-branch frequency–spatial enhanced backbone. This branch mainly consists of AFDC and MFFWBlock, as shown in **Figures 2**, **3**, respectively. AFDC enhances high-frequency edge and fine-grained texture representation during downsampling, while MFFWBlock further fuses and models multiscale frequency-domain information.

**Figure 2:**
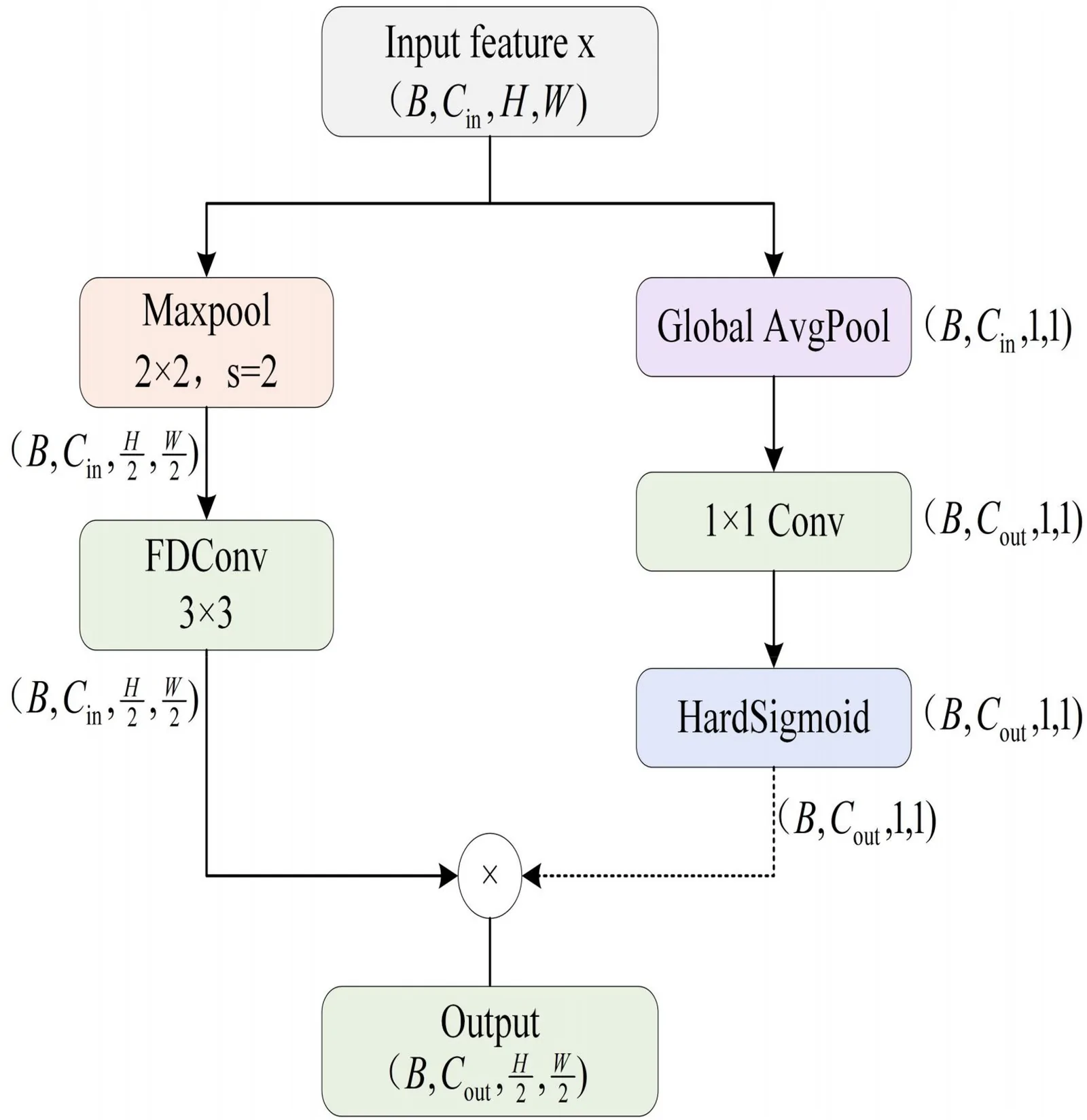
AFDC module.

**Figure 3:**
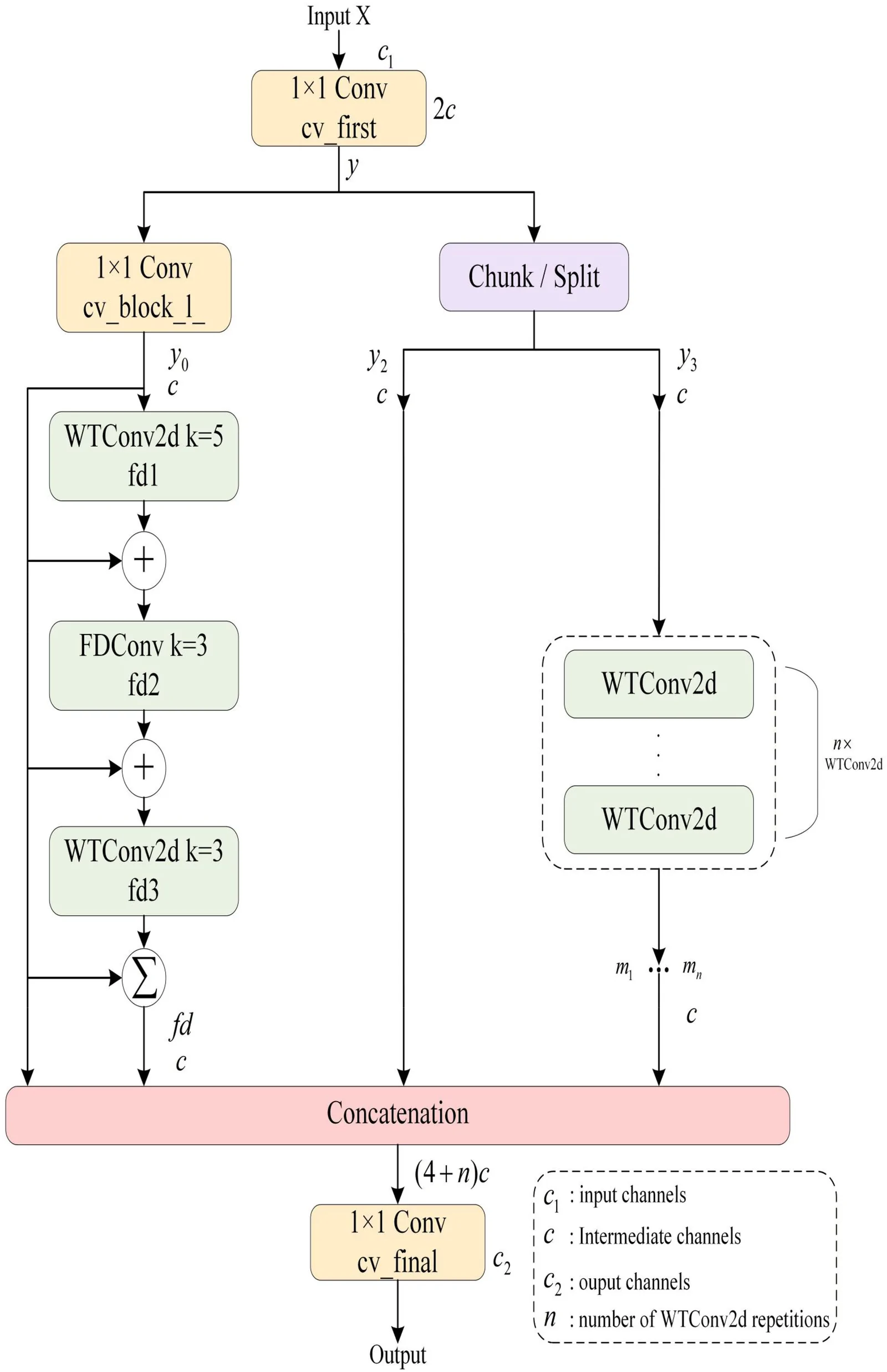
MFFWBlock module.

#### 3.2.1 AFDC module

Given an input feature $X\in {\mathbb{R}}^{B\times C_{\text{in}}\times H\times W}$, AFDC first uses max pooling for spatial downsampling and then applies FDConv (Chen et al., 2025) to enhance the frequency-domain response, obtaining the downsampled frequency-enhanced feature *X_d_*, as shown in Equation 1. Meanwhile, the input feature *X* passes through global average pooling, a 1×1 convolution, and a HardSigmoid function to generate the channel attention weight *A*, as shown in Equation 2. Finally, AFDC uses the channel attention weight to modulate the frequency-enhanced feature and obtains the output feature *Y*, as shown in Equation 3:

(1) $$X_{d}={\text{FDC}}_{{\mathrm{conv}}_{3\times 3}}\left(\mathrm{Max}{\text{Pool}}_{2\times 2}\left(X\right)\right)$$

(2) $$A=\text{Hardsigmoid}\left({\mathrm{Conv}}_{1\times 1}\left(\text{GAP}\left(X\right)\right)\right)$$

(3) $$Y=X_{d}⊙A$$

where ⊙ denotes element-wise multiplication, $A\in {\mathbb{R}}^{B\times C_{\text{out}}\times 1\times 1}$. As shown in Equations 1–3, AFDC introduces FDConv to enhance edge and texture responses while reducing the spatial resolution of the feature map. The channel attention further highlights effective channel features, which helps reduce the interference of background noise with lesion detail representation.

#### 3.2.2 MFFWBlock

Given an input feature $X\in {\mathbb{R}}^{B\times C_{1}\times H\times W}$, MFFWBlock first uses a 1×1 convolution for channel expansion and obtains the intermediate feature $y\in {\mathbb{R}}^{B\times 2C\times H\times W}$. Next, *y* is divided into two paths. One path passes through a 1 × 1 convolution to obtain the input feature *y*_0_ of the main frequency-enhancement path. The other path is split along the channel dimension into two sub-features, *y*_2_ and $y_{3}\in {\mathbb{R}}^{B\times C\times H\times W}$, as shown in Equation 1. The main path takes *y*_0_ as input and sequentially applies a 5 × 5 wavelet convolution, a 3 × 3 frequency-domain convolution, and a 3 × 3 wavelet convolution to extract frequency-domain texture responses at different scales, as shown in Equations 5–7. Feature *y*_3_ is fed into consecutive WTConv2d modules to generate multilevel wavelet texture features *m_i_*, as shown in Equation 8, where $m_{0}=y_{3}$, and *n* denotes the number of repeated WTConv2d modules. Finally, the module concatenates features from different branches along the channel dimension and uses a 1 × 1 convolution for compression and fusion, as shown in Equation 9.

(4) $$\left[y_{2},y_{3}\right]=\text{Chunk}\left(y,2,\dim=1\right)$$

(5) $$f_{d1}=\text{WT}{\mathrm{Conv}}_{5\times 5}\left(y_{0}\right)$$

(6) $$f_{d2}=\text{FD}{\mathrm{Conv}}_{3\times 3}\left(f_{d1}+y_{0}\right)$$

(7) $$f_{d3}=\text{WT}{\text{Conv}}_{3\times 3}\left(f_{d2}+f_{d1}\right)$$

(8) $$m_{i}=\text{WT}{\mathrm{Conv}}_{i}\left(m_{i-1}\right),\;i=1,2,\ldots ,n$$

(9) $$Y_{\text{MFFW}}={\mathrm{Conv}}_{1\times 1}^{\text{final}}\left(\mathrm{Concat}\left(y_{0},f_{d1},f_{d2},f_{d3},m_{1},\ldots ,m_{n}\right)\right)$$

As shown in Equations 4–9, MFFWBlock uses wavelet convolution and frequency-domain convolution to extract local edges, fine-grained textures, and multiscale frequency-domain responses simultaneously. Combined with the downsampling frequency enhancement of AFDC, the frequency-domain enhancement branch can provide more discriminative disease feature representations for subsequent detection.

### 3.3 Spatial enhancement branch

Although frequency-domain features help describe lesion edges and detailed textures, eggplant fruits in real field environments are often affected by leaf occlusion, branch overlap, and complex background interference. Relying only on local texture information may lead to false detections or missed detections. Therefore, this study further builds a spatial enhancement branch to capture spatial dependencies between disease and pest regions and their surrounding fruits, leaves, branches, and backgrounds. This branch mainly consists of VisionClueMerge and G6MambaLayer.

#### 3.3.1 VisionClueMerge

Traditional downsampling usually uses strided convolution or pooling to directly reduce the size of feature maps. Although this operation can reduce computational cost, it may also cause the loss of local spatial cues, which is particularly unfavorable for preserving weak lesions and blurred boundary regions. To address this problem, this study introduces VisionClueMerge (Wang et al., 2025b) to replace traditional downsampling operations, and its structure is shown in **Figure 4**. VisionClueMerge rearranges adjacent spatial features within each 2 × 2 neighborhood into the channel dimension, as shown in Equations 10–13, and uses a 1 × 1 convolution for channel mapping. In this way, it reduces the spatial resolution of the feature map while preserving local spatial cues. This design helps reduce detail loss during downsampling and provides more complete local features for subsequent spatial dependency modeling.

**Figure 4:**
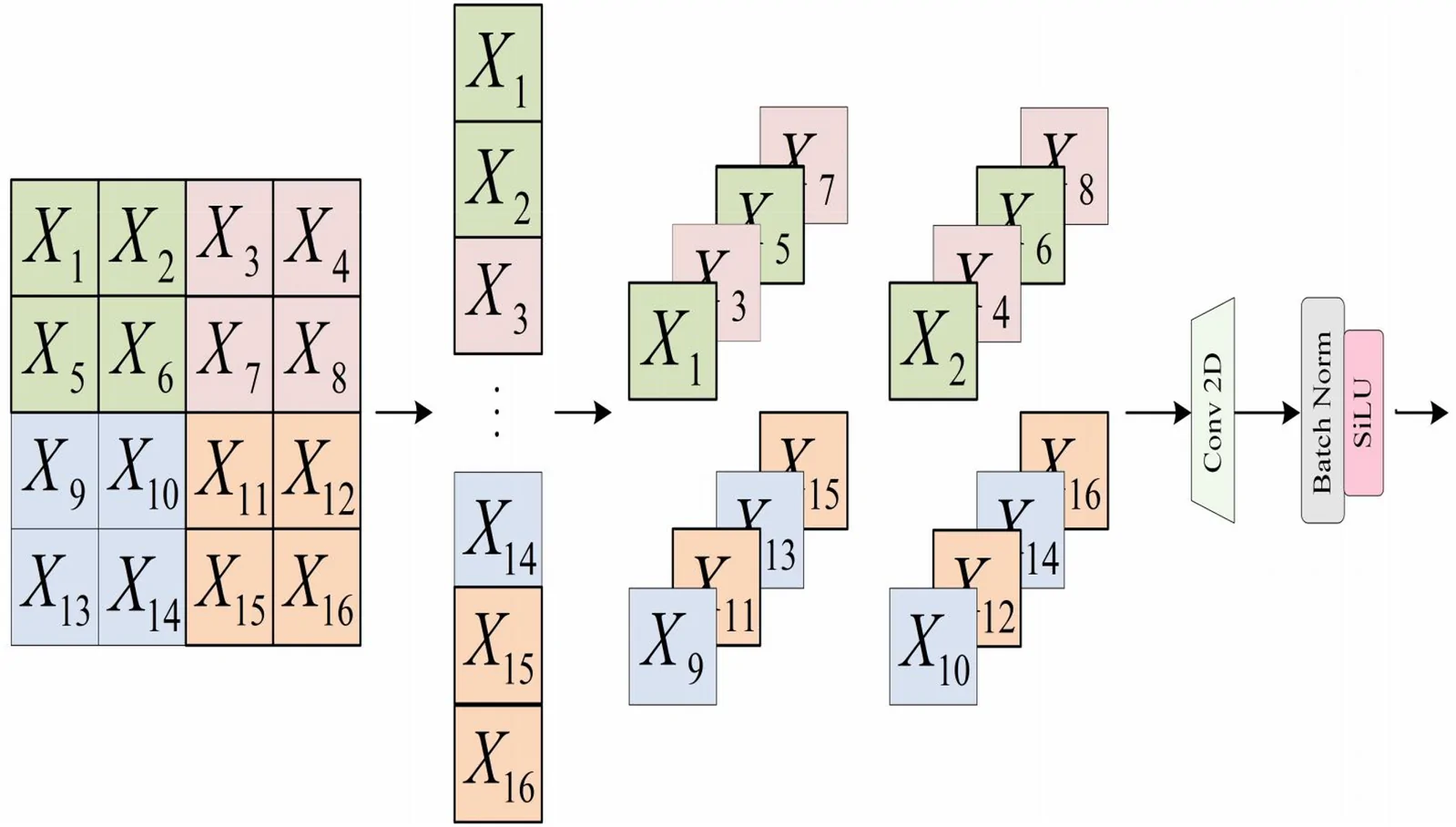
VisionClueMerge module.

(10) $$X_{1}=X\left[:,:,0::2,0::2\right]$$

(11) $$X_{2}=X\left[:,:,1::2,0::2\right]$$

(12) $$X_{3}=X\left[:,:,0::2,1::2\right]$$

(13) $$X_{4}=X\left[:,:,1::2,1::2\right]$$

#### 3.3.2 G6MambaLayer

Existing VMamba (Liu et al., 2024) usually adopts four-direction scanning for visual state space modeling, which can capture long-range spatial dependencies in images to some extent; some studies (Safari et al., 2026) have shown that using only horizontal and vertical scanning can separate the central pixel from its diagonal neighbors, thereby limiting the modeling of long-range spatial dependencies. However, in agricultural planting scenes, fruits, lesions, leaves, branches, and backgrounds often show irregular spatial distributions. Disease and pest regions may also extend along diagonal edges, branch directions, or fruit contours. Feature propagation that relies only on horizontal and vertical directions makes it difficult to fully model such irregular spatial relationships. RS-Mamba (Shi and Wang, 2025) introduces diagonal scanning to construct an omnidirectional state space with eight scanning sequences; however, despite capturing richer features, this approach incurs higher computational costs and slower processing (Xu et al., 2024). Therefore, this study designs G6MambaLayer, as shown in **Figure 5**. Based on the four-direction scanning strategy, G6MambaLayer further introduces diagonal modeling paths and constructs six selective scanning paths to enhance the model’s ability to represent complex spatial contextual relationships.

**Figure 5:**
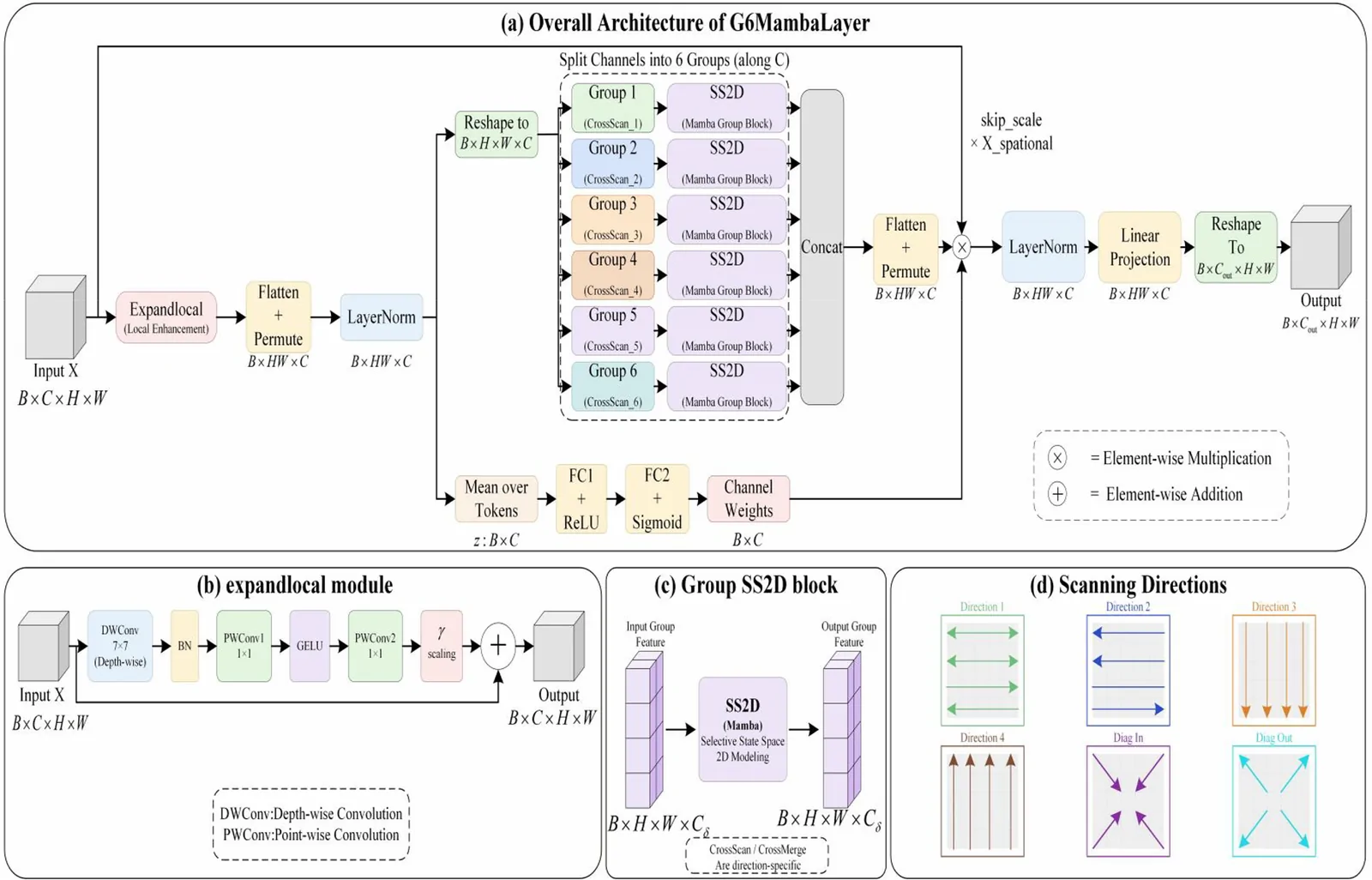
Overview of G6MambaLayer. The Architecture of G6MamberLayer is labeled **(a)**. The expandlocal module **(b)** and the SS2D **(c)** are the main modules of G6MamberLayer. The **(d)** shows six selective scanning paths of G6MamberLayer.

Given an input feature *X*, the local expansion module first enhances neighborhood texture information, as shown in Equation 14. The enhanced feature is flattened into a sequence form and normalized according to Equation 15. To further highlight key channel information, this study performs average aggregation on the normalized sequence features along the token dimension to obtain a global channel descriptor. A two-layer fully connected network is then used to generate channel recalibration weights, as shown in Equations 16, 17:

(14) $$X_{\mathrm{local}}=X+\gamma \cdot \text{PW}\mathrm{Conv}2\left(\text{GELU}\left(\text{PW}\mathrm{Conv}1\left(\text{BN}\left(\text{DW}{\mathrm{Conv}}_{7\times 7}\left(X\right)\right)\right)\right)\right)$$

(15) $$X_{\text{seq}}=\text{LN}\left(\text{Flatten}\left(X_{\text{local}}\right)\right)$$

(16) $$Z=\text{Mean}\left(X_{\mathrm{seq}},\text{dim}=\text{token}\right)$$

(17) s=Sigmoid(FC2(ReLU(FC1(Z))))

where $\text{DW}{\mathrm{Conv}}_{7\times 7}$ denotes a 7 × 7 depth-wise convolution, PWConv denotes point-wise convolution, and γ is a learnable scaling parameter. $z\in {\mathbb{R}}^{B\times C}$ represents the global channel descriptor obtained by average aggregation across all spatial tokens, and $s\in {\mathbb{R}}^{B\times C}$ represents the channel recalibration weight. Subsequently, the sequence feature is reshaped back into spatial form and split into six groups along the channel dimension, as shown in Equation 18. The six groups of features are fed into SS2D scanning branches with different directions to model spatial dependencies in the horizontal, vertical, and diagonal directions, as shown in Equation 19. Next, the output features from the six directions are concatenated to obtain the multidirectionally spatially enhanced feature, as shown in Equation 20. Finally, skip_scale is introduced to perform element-wise modulation with the original spatial feature, and the channel weight *s* further recalibrates the feature. The output is then obtained through LayerNorm and linear projection, as shown in Equation 21, where $Y\in R^{B\times C_{\text{out}}\times H\times W}$.

(18) $$\left[X_{1},X_{2},X_{3},X_{4},X_{5},X_{6}\right]=\text{Split}\left(X_{\mathrm{spatial}},6,\dim=C\right)$$

(19) $$M_{i}={SS2D}_{i}\left(X_{i}\right),\;i=1,2,\ldots ,6$$

(20) $$M=\mathrm{Concat}\left(M_{1},M_{2},M_{3},M_{4},M_{5},M_{6}\right)$$

(21) $$Y=\text{Reshape}\left(\text{Linear}\left(\text{LN}\left(\text{Flatten}\left(M\cdot \text{skip}\_\text{scale}\cdot X_{\text{spatial}}\right)\cdot s\right)\right)\right)$$

G6MambaLayer expands the range of spatial information interaction and more fully models the contextual relationships between disease and pest regions and surrounding fruits, leaves, branches, and backgrounds. This improves the model’s ability to represent occluded targets, small lesions, and complex background interference.

### 3.4 Frequency–spatial feature fusion enhancement module

The frequency-domain branch focuses on capturing lesion edges, high-frequency textures, and local abnormal responses, while the spatial branch focuses on modeling target structures, occlusion relationships, and spatial context. These two types of features are complementary. To effectively fuse frequency-domain detail information and spatial semantic information, this study designs Enhanced Gated Multiattention Fusion (EGMuF) for adaptive frequency–spatial feature fusion, as shown in **Figure 6**. Unlike direct concatenation, EGMuF selectively fuses the two types of features through a multilevel attention mechanism. For small lesions, weak edges, and pest bite marks, frequency-domain features can provide more evident texture responses. For targets affected by occlusion, similar backgrounds, and large-scale variations, spatial features can provide more stable contextual information. Through this module, the model can dynamically integrate effective features under different disease and pest scenarios, thereby improving detection robustness in complex scenes.

**Figure 6:**
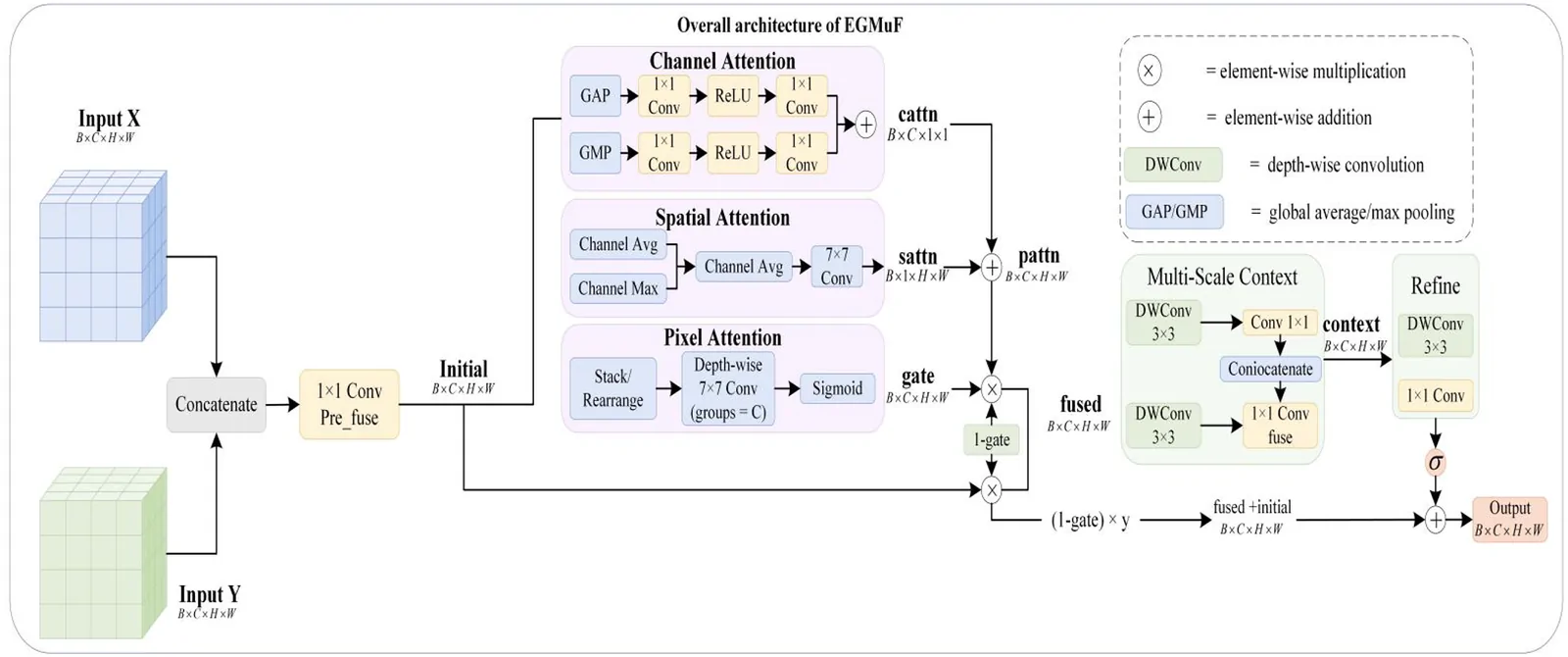
Overview of the EGMuF module.

For the given frequency-branch feature *X* and spatial-branch feature *Y*, EGMuF first concatenates them along the channel dimension and then applies a 1 × 1 convolution to obtain the initial fused feature. Next, EGMuF constructs channel attention, spatial attention, and pixel attention. Channel attention highlights informative feature channels, spatial attention locates key disease regions, and pixel attention generates fine-grained gating weights. The channel attention branch extracts channel descriptors through global average pooling and global max pooling, and then generates channel attention using 1 × 1 convolutions. The spatial attention branch obtains spatial descriptors from the channel-wise average pooling response and channel-wise max pooling response, and then uses a 7 × 7 convolution to generate spatial attention. The channel attention and spatial attention are then added together. In the pixel attention branch, EGMuF stacks and rearranges the input features and generates a pixel-level gating map through a 7 × 7 depth-wise convolution. To preserve the initial fusion information, the module introduces a residual connection and performs adaptive weighted fusion. It then uses 3 × 3 and 5 × 5 depth-wise convolution branches to extract contextual features with different receptive fields and applies 1 × 1 convolutions for channel mapping. Finally, the module further adjusts the fused feature through an output refinement branch and introduces a learnable residual coefficient to obtain the final output.

## 4 Dataset construction and evaluation metrics

### 4.1 Dataset construction

This study uses a public eggplant fruit disease and pest dataset from the Roboflow platform (UNIVERSITY, 2023) for the main experiments. The dataset contains 3,250 images. We divide the dataset into training and validation sets at a ratio of 8:2, with 2,610 images in the training set and 640 images in the validation set. The annotated categories include four classes: FruitRot, FruitBorer, MelonThrips, and Healthy. Statistics for individual class samples are shown in **Table 1**. Representative samples are marked in red in **Figure 7**. Unlike disease images collected under laboratory conditions, the samples in this dataset are closer to natural growing environments. The images contain challenges such as leaf and branch occlusion, cluttered backgrounds, direct strong light, local shadows, and blurred lesion boundaries, as shown in **Figure 8**. These factors can weaken edge and texture features in disease and pest regions and increase the difficulty of distinguishing targets from the background. In **Table 1**, the training set contains the largest and most similar sample sizes for melon thrips (794 images) and healthy samples (789 images), followed by fruit borers (599 images) and fruit rot (428 images), indicating a certain degree of class imbalance. To mitigate the risk of underfitting caused by the scarcity of minority class samples, geometric and photometric transformation data augmentation methods, such as random rotation, horizontal and vertical flipping, brightness and contrast perturbation, random cropping, and scaling, were applied to the fruit rot class to expand the sample size and enhance intraclass feature diversity. This improves the model’s generalization ability for this disease class and mitigates the negative impact of class imbalance on training performance.

**Table 1:** The images and instances in the eggplant dataset.

Training set			Validation set		
Class	Images	Instances	Class	Images	Instances
FruitRot	428	1,216	FruitRot	139	495
FruitBorer	599	1,084	FruitBorer	191	261
MelonThrips	794	1,224	MelonThrips	205	356
Healthy	789	1,332	Healthy	185	334
Total	2,610	4,856	Total	640	1,446

**Figure 7:**
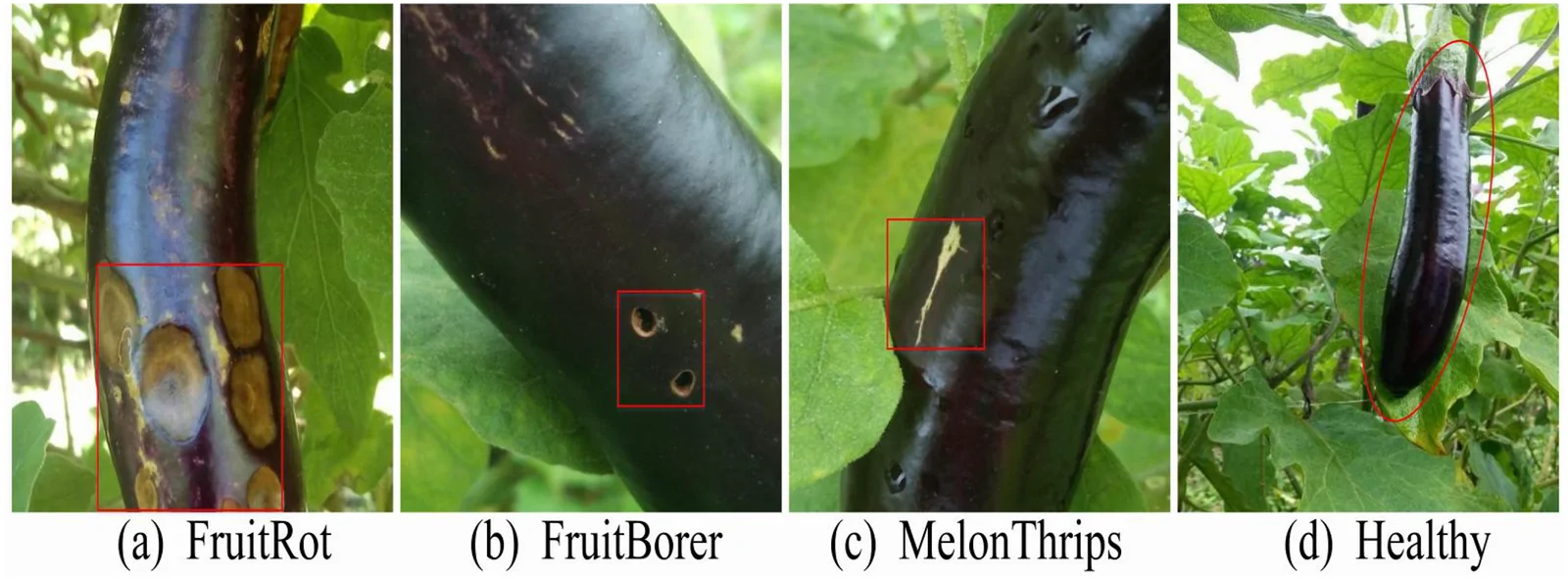
Examples of eggplant fruit diseases and pests: **(a)** FruitRot, **(b)** FruitBorer, **(c)** MelonThrips, and **(d)** Healthy.

**Figure 8:**
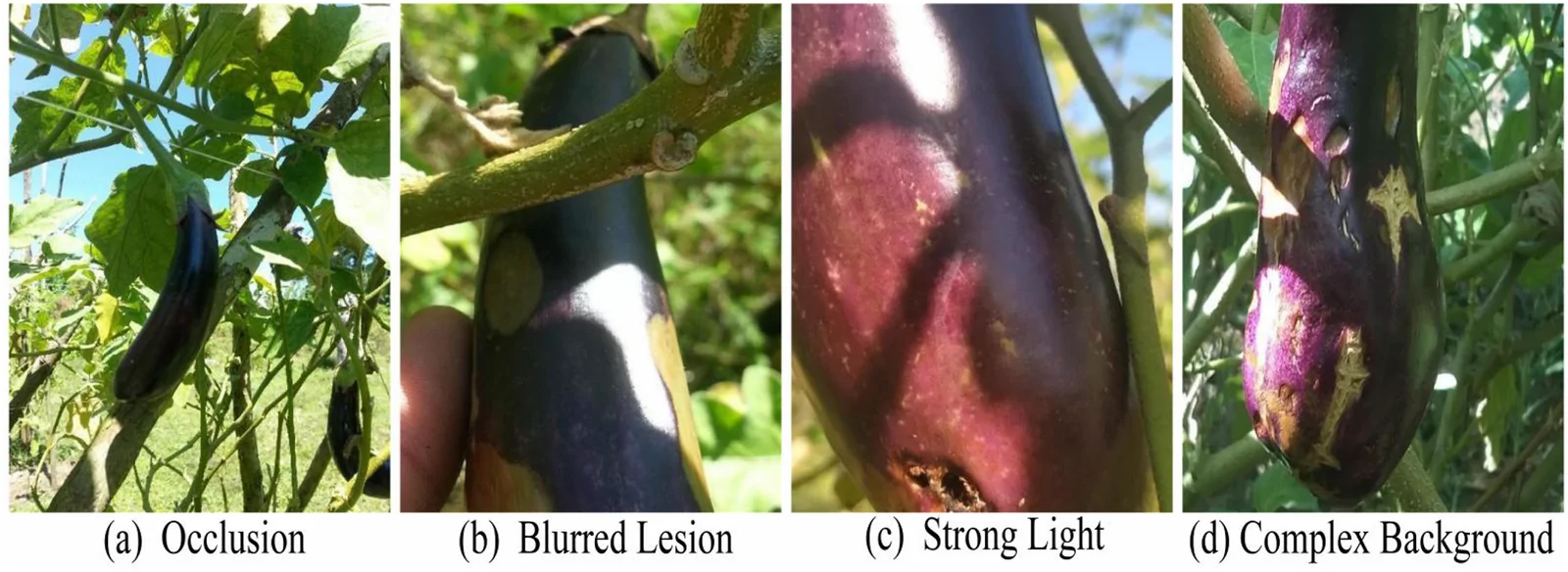
Typical samples from the eggplant dataset under natural growing conditions: **(a)** occlusion, **(b)** blurred lesion, **(c)** strong light, and **(d)** complex background.

In addition, to verify the adaptability of the model to other plant disease detection tasks, this study further selects the other publicly available PlantDoc (Singh et al., 2020) dataset as an external validation dataset. PlantDoc contains 2,598 real-scene images, covering 13 plant species and 27 healthy or diseased categories. Compared with a single-crop dataset, PlantDoc shows greater diversity in crop types, disease appearances, and background environments, making it suitable for evaluating the model’s generalization ability in cross-dataset scenarios. The dataset was divided into training, testing, and validation sets at a ratio of 7:1:2. Representative images from this dataset are shown in **Figure 9**.

**Figure 9:**
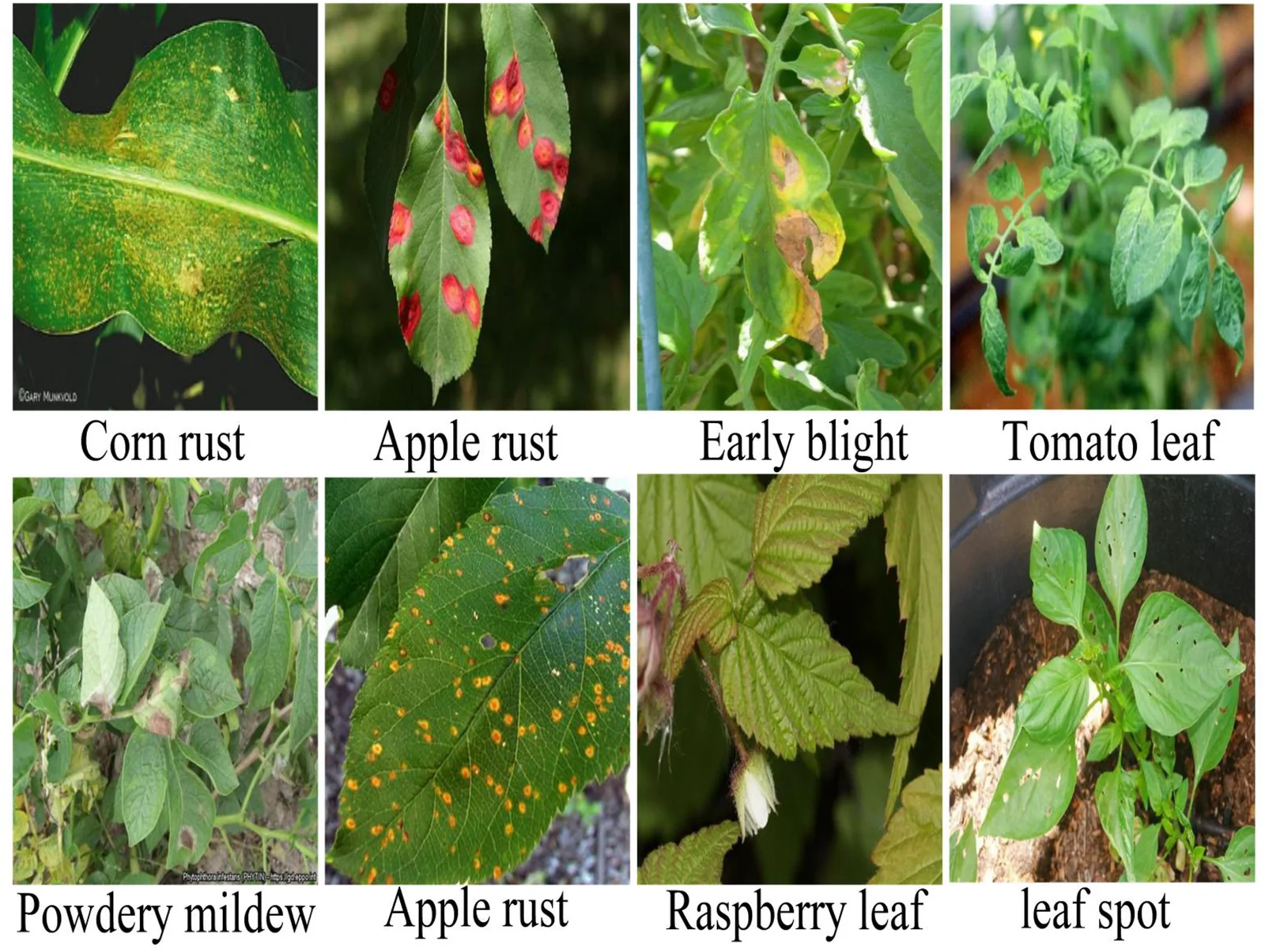
Representative examples from the PlantDoc dataset.

The Eggplant dataset was obtained from Roboflow Universe, whereas the PlantDoc dataset was obtained from its publicly available Kaggle repository. The publicly available Roboflow page of the Eggplant dataset does not report a reproducible preprocessing or augmentation configuration, and the Kaggle repository of PlantDoc does not specify an additional platform-level augmentation policy. Therefore, the source datasets were treated as the original annotated datasets rather than as independently augmented datasets. It should be noted that the original PlantDoc benchmark applied random horizontal flipping and random SSD cropping during object-detection training. In our experiments, however, all compared models were trained using the same augmentation pipeline to minimize the influence of model-specific data processing and ensure a fair comparison.

### 4.2 Evaluation metrics

To comprehensively evaluate the performance of the proposed model in eggplant fruit disease and pest detection, this study evaluates performance from two aspects: detection accuracy and model complexity. For detection accuracy, Precision (P), Recall (R), mAP_50_, and mAP_50:95_ are selected as the main metrics. For model complexity, Parameters and GFLOPs are used to measure model size and computational cost. To improve the practical application value of the model, Parameters and GFLOPs are controlled while maintaining detection accuracy. The main formulas of these metrics are shown in Equations 22–25, where TP denotes the number of correctly detected targets, FP denotes the number of background or healthy regions incorrectly classified as disease or pest targets, and FN denotes the number of real disease or pest targets that are not detected; *N* is the number of classes.

(22) $$\text{Precision}=\frac{\text{TP}}{\text{TP}+\text{FP}}$$

(23) $$\text{Recall}=\frac{\text{TP}}{\text{TP}+\text{FN}}$$

(24) $$\text{AP}=\int_{0}^{1}\mathrm{P(R)d}R$$

(25) $$\text{mAP}=\frac{1}{N}\sum_{i=1}^{N}{\text{AP}}_{i}$$

## 5 Experiments

### 5.1 Experimental environment

To reduce the influence of differences in experimental environments and parameter settings on model performance comparison, all experiments in this study use the same hardware equipment, software environment, and training strategy. All models are trained and tested under the same data split, input size, number of training epochs, and optimizer settings. The detailed experimental environment and main parameter settings are shown in **Table 2**. To ensure a fair and reproducible comparison, all models are trained and evaluated using a unified experimental protocol. PlantDoc and the Eggplant dataset are publicly available datasets. For each dataset, a fixed training, validation, and test split was established before training and remained unchanged for all models. All models were initialized with the same pretrained weights, and no additional task-specific pretraining was introduced. Here, FreqE denotes the frequency-domain enhancement branch, SpaE denotes the spatial enhancement branch, and EGMuF denotes the frequency–spatial feature fusion enhancement module.

**Table 2:** Experimental environment and parameter settings.

Hardware configuration		Training parameters	
Type	Value	Parameter	Value
OS	Linux	Epoch	300
GPU	RTX4090 24G	*Lr*_0_	0.0001
CPU	i9 13900k	Batch size	8
CUDA	12.1	Optimizer	AdamW
Cudnn	8.9.5	Workers	8
Worker	8	Momentum	0.9
PyTorch-wavelets	1.3.0	Weight_Decay	0.0001
Selective_Scan	0.0.2	imgsz	640
Grad-Cam	1.5.4	*Lrf*	1.0

### 5.2 Ablation experiments

To verify the effectiveness of different structural designs in the proposed model and analyze the contribution of each module, this study uses RT-DETR-R18 as the baseline model. FreqE, SpaE, and EGMuF are introduced separately and in different combinations. Ablation experiments are conducted on the public Eggplant dataset and PlantDoc dataset.

#### 5.2.1 Ablation experiments on the Eggplant dataset

##### 5.2.1.1 Performance metrics of ablation experiments

To verify the impact of each key module on model performance, ablation experiments are conducted on the Eggplant dataset. **Table 3** reports the overall detection performance of different module combinations in terms of Precision, Recall, mAP_50_, mAP_50:95_, Parameters, GFLOPs, and FPS, where “✓” and “×” indicate whether the corresponding module was included or excluded. To further analyze the influence of each module on different target categories, Precision, Recall, mAP_50_, and mAP_50:95_ are calculated for four typical classes, namely FruitRot, FruitBorer, Healthy, and MelonThrips. The results are shown in **Figure 10**.

**Table 3:** Ablation results of key modules in FSF-RTDETR on the Eggplant dataset.

Baseline	FreqE	SpaE	EGMFusion	P (%)	R (%)	mAP_50_ (%)	mAP_50:95_ (%)	Params	GFLOPs	FPS
✓	×	×	×	79.8	69.3	74.6	46.0	19.87M	57.0G	**227.27**
✓	✓	×	×	80.2	70.5	75.6	48.3	22.96M	**30.2G**	133.33
✓	×	✓	×	81.2	69.7	75.8	49.2	18.03M	35.2G	126.58
✓	×	×	✓	79.2	70.4	76.7	48.0	25.54M	64.5G	128.21
✓	✓	✓	×	79.7	71.3	76.8	49.5	**17.33M**	38.8G	83.33
✓	✓	×	✓	75.7	70.0	77.7	49.1	29.36M	75.9G	101.01
✓	×	✓	✓	80.7	70.1	78.4	50.2	28.67M	82.8G	93.46
✓	✓	✓	✓	**81.5**	**71.3**	**78.8**	**50.7**	21.54M	51.7G	104.07

The best results are shown in bold.

**Figure 10:**
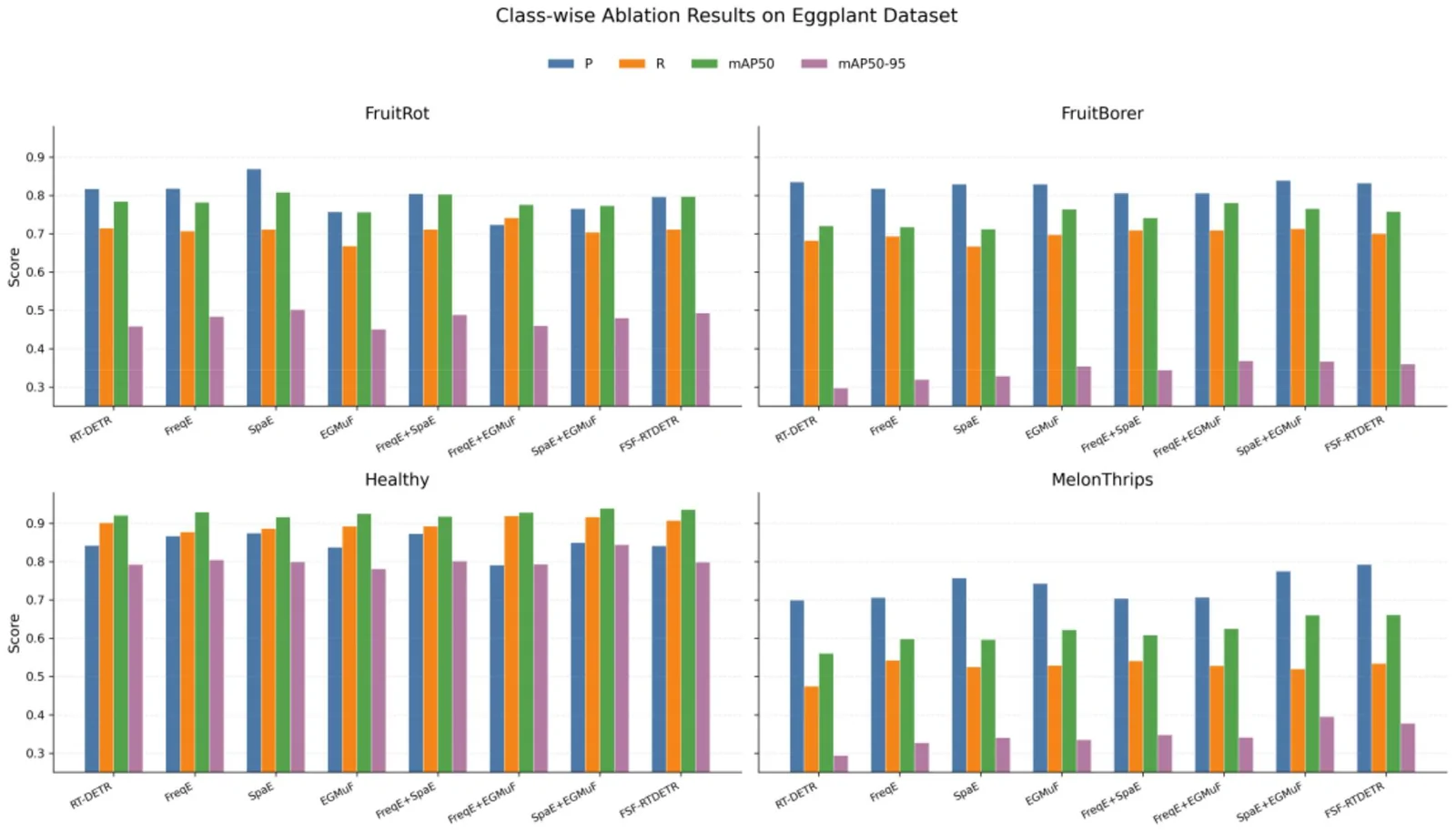
Performance of FSF-RTDETR ablation models on categories in the Eggplant dataset.

As shown in **Table 3**, both the frequency enhancement branch and the spatial enhancement branch improve mAP_50_ and mAP_50:95_ when introduced separately. This indicates that both types of feature enhancement can improve disease and pest detection performance. The spatial enhancement branch brings more evident gains in Precision and mAP_50:95_, suggesting that spatial dependency modeling helps strengthen target representation under complex backgrounds. After introducing EGMuF, mAP_50_ increases further, showing that frequency–spatial feature fusion can enhance effective feature representation. Finally, FSF-RTDETR achieves Precision, Recall, mAP_50_, and mAP_50:95_ of 81.5%, 71.3%, 78.8%, and 50.7%, respectively, outperforming the baseline by 1.7, 2.0, 4.2, and 4.7 percentage points. **Figure 10** shows that the frequency enhancement branch is more beneficial for fine-grained disease and pest categories such as FruitRot, FruitBorer, and MelonThrips, while the spatial enhancement branch improves detection stability under occlusion and complex backgrounds.

To evaluate the individual contributions and combined effect of AFDC and MFFWBlock in the FreqE branch, a component-wise ablation study was conducted under identical experimental settings. Four configurations were evaluated, and their mAP_50_, mAP_75_, and mAP_50:95_ results are reported in **Table 4**.

**Table 4:** Ablation results of the FreqE branch in FSF-RTDETR on the Eggplant dataset.

Baseline	AFDC	MFFWBlock	mAP_50_ (%)	mAP_75_ (%)	mAP_50:95_ (%)	p-value (%)	R (%)
✓	×	×	74.6	66.7	46.0	66.5	53.1
✓	✓	×	75.8	65.2	45.1	65.4	55.2
✓	×	✓	**76.3**	67.3	45.7	64.1	57.6
✓	✓	✓	75.6	**68.6**	**48.3**	**64.3**	**61.8**

The best results are shown in bold.

As shown in **Table 4**, the complete FreqE branch improves mAP_50_, mAP_75_, and mAP_50:95_ by 1.0, 1.9, and 2.3 percentage points over the baseline, respectively. Although each module shows different performance when used alone, their combination achieves the highest mAP_75_ and mAP_50:95_, demonstrating improved localization under stricter IoU thresholds.

To evaluate the individual contributions and combined effect of VisionClueMerge and G6MambaLayer in the SpaE branch, a component-wise ablation study was conducted under identical experimental settings. Four configurations were evaluated, and their mAP_50_, mAP_75_, and mAP_50:95_ results are reported in **Table 5**.

**Table 5:** Ablation results of the SpaE branch in FSF-RTDETR on the Eggplant dataset.

Baseline	VisionClueMerge	G6MambaLayer	mAP_50_ (%)	mAP_75_ (%)	mAP_50:95_ (%)	*p*-value (%)	*R* (%)
✓	×	×	74.6	66.7	46.0	**66.5**	53.1
✓	✓	×	75.0	67.9	46.5	63.4	54.8
✓	×	✓	75.3	68.1	48.4	62.7	56.1
✓	✓	✓	**75.8**	**69.2**	**50.7**	60.4	**57.8**

The best results are shown in bold.

As reported in **Table 5**, VisionClueMerge slightly improves detection by retaining local information during downsampling, whereas G6MambaLayer brings greater gains through multidirectional feature modeling. Combining both modules yields 69.2% mAP_75_ and 50.7% mAP_50:95_, exceeding the baseline by 2.5 and 4.7 percentage points. Despite a slightly lower mAP_50_ than G6MambaLayer alone, the combined model performs better at stricter IoU thresholds, indicating more accurate localization.

##### 5.2.1.2 Visualization of ablation experiments

To further visually compare the influence of different modules on detection results, FruitRot, FruitBorer, Healthy, and multidisease/pest cooccurrence scenes are selected as representative samples for visualization analysis of different ablation models. Here, mixed disease denotes a complex scene in which multiple disease or pest targets appear in the same image. **Figures 11**, **12** show comparisons of the detections from different ablation models and the Gradient-weighted Class Activation Mapping (Grad-CAM) visualization results on eggplant fruit disease and pest samples, respectively.

**Figure 11:**
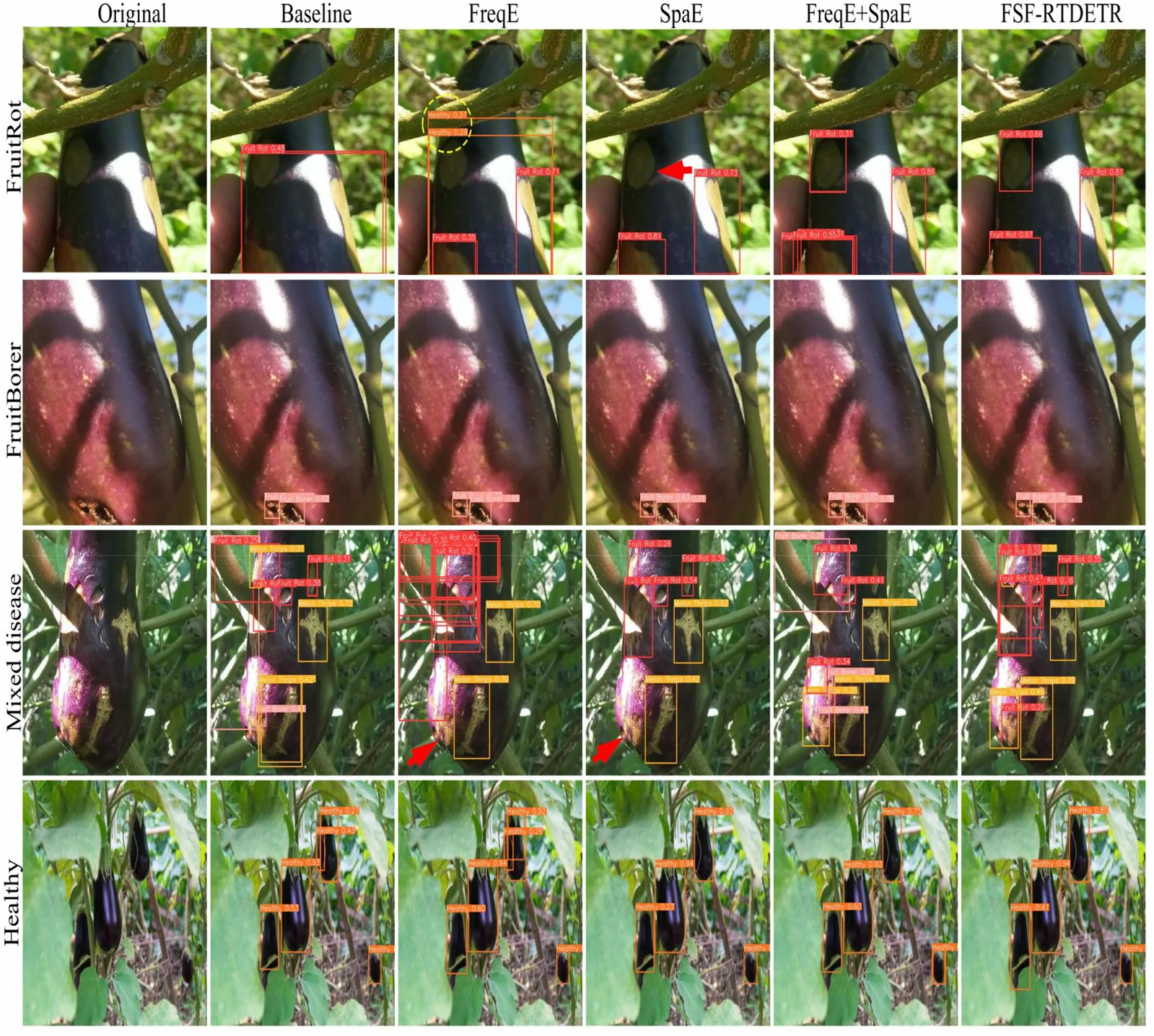
Visualization of detection results of different fusion modules on the dataset. Yellow dashed circles indicate false detections, and red arrows indicate missed detections.

**Figure 12:**
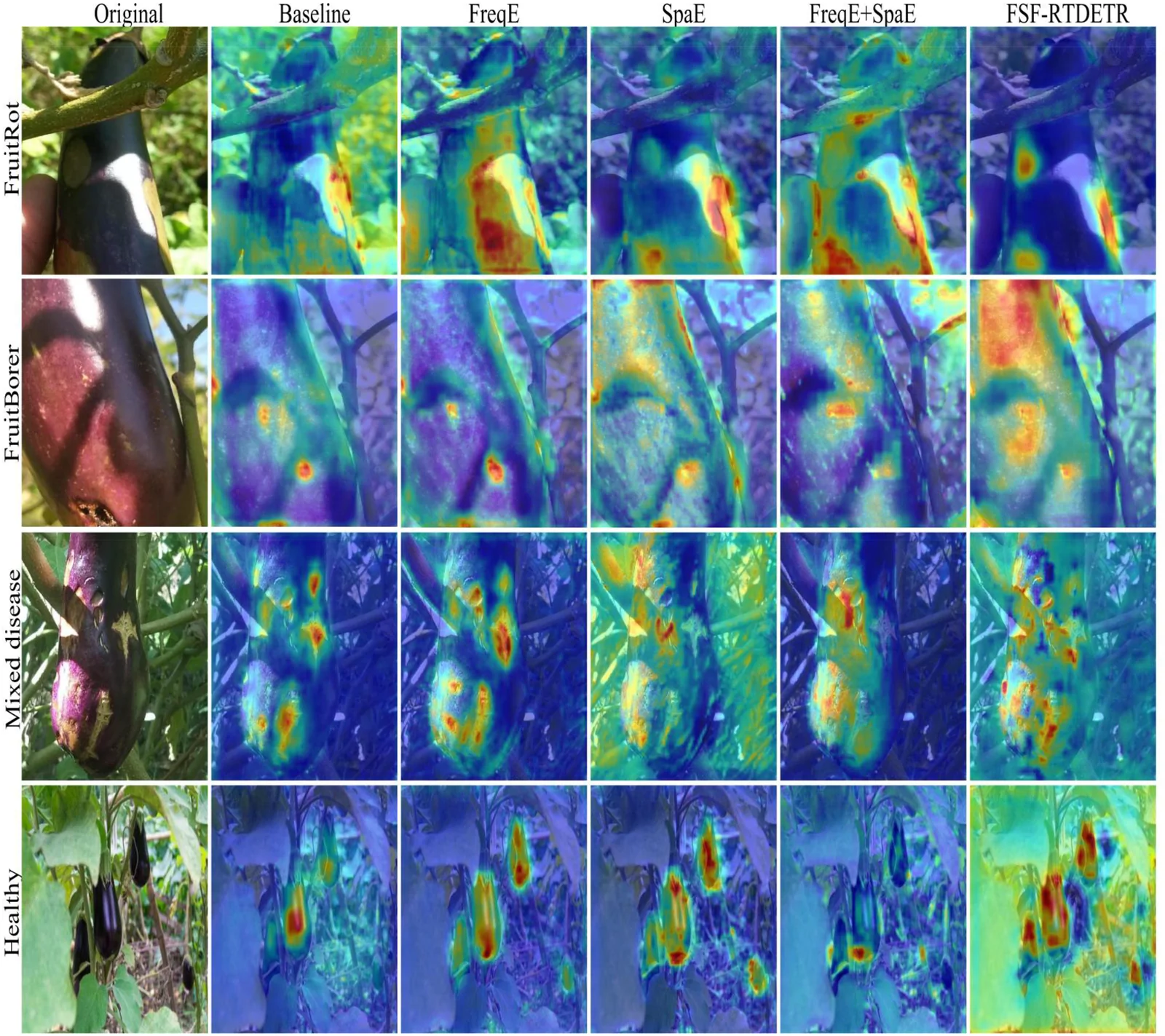
Grad-CAM of ablation modules in FSF-RTDETR on the Eggplant dataset.

As shown in **Figure 11**, the baseline model tends to suffer from missed detections, false detections, or incomplete localization in complex backgrounds, small-lesion regions, and multidisease and pest scenarios. FreqE enhances the response to local lesion textures and edge regions, but false detections may still occur in areas with strong reflections or complex textures. SpaE improves localization stability under occlusion by modeling spatial structures and contextual information. After combining FreqE and SpaE, the model more completely covers the main disease regions. Overall, FSF-RTDETR produces more stable detection results in most typical scenes. This further confirms the complementary effect of frequency-detail enhancement and spatial semantic modeling. As shown in **Figure 12**, the baseline model shows scattered responses in scenes with small lesions, occlusion, and multiple coexisting diseases or pests. After introducing FreqE, the model strengthens its responses to lesion edges, fruit surface textures, and local abnormal regions. Compared with the baseline, the highlighted areas in the FreqE column are more compact and contain less background noise, indicating that the frequency features effectively suppress irrelevant background interference. In scenarios with mixed diseases, the frequency branch can simultaneously respond to diseased areas on two eggplants, demonstrating its ability to locate multiple small lesions. It maintains moderate contour highlights on the healthy eggplant, but it may still activate some irrelevant high-frequency areas under strong light or complex backgrounds. SpaE improves the model’s perception of fruit bodies and occluded regions through spatial context modeling. FreqE + SpaE further preserves both local details and spatial structures. The proposed FSF-RTDETR maintains stronger responses in the main disease and pest regions, indicating the complementarity of frequency-detail enhancement and spatial semantic modeling.

#### 5.2.2 Ablation experiments on the PlantDoc dataset

Compared with the eggplant fruit dataset, the PlantDoc dataset contains more crop species, larger variations in disease appearance, and more complex image backgrounds and annotation scales. In some samples, the bounding boxes cover whole leaves or large disease regions, which places higher demands on the model’s cross-category recognition ability and adaptability to complex backgrounds.

##### 5.2.2.1 Performance metrics of ablation experiments

**Table 6** reports the overall detection performance of different module combinations and is used to evaluate the impact of each module on cross-dataset detection results. **Figure 13** further shows the class-wise performance changes of different ablation models on four typical classes: corn rust, early blight, powdery mildew, and leaf spot. These four classes correspond to fine-grained rust-like textures, large irregular lesions, powdery disease textures, and leaf spot diseases under complex backgrounds, respectively. They effectively reflect the detection difficulties caused by different disease appearances and scene conditions in the PlantDoc dataset.

**Table 6:** Ablation results of key modules in FSF-RTDETR on the PlantDoc dataset.

Baseline	FreqE	SpaE	EGMuF	p-value (%)	R (%)	mAP_50_ (%)	mAP_50:95_ (%)	Params	GFLOPs	FPS
✓	×	×	×	66.5	53.1	53.7	42.8	19.87M	57.0G	**101.01**
✓	✓	×	×	64.3	61.8	61.2	49.7	**17.96M**	**27.2G**	73.53
✓	×	✓	×	60.4	57.8	59.1	47.8	23.03M	35.2G	69.93
✓	×	×	✓	63.2	69.0	68.9	55.5	35.54M	104.5G	76.92
✓	✓	✓	×	63.3	67.3	67.7	54.3	17.33M	38.8G	55.25
✓	✓	×	✓	65.4	69.6	68.5	56.4	29.36M	75.9G	68.03
✓	×	✓	✓	66.0	71.8	68.7	57.2	28.67M	82.8G	64.10
✓	✓	✓	✓	**68.2**	**69.2**	**70.8**	**58.2**	21.54M	51.7G	81.02

The best results are shown in bold.

**Figure 13:**
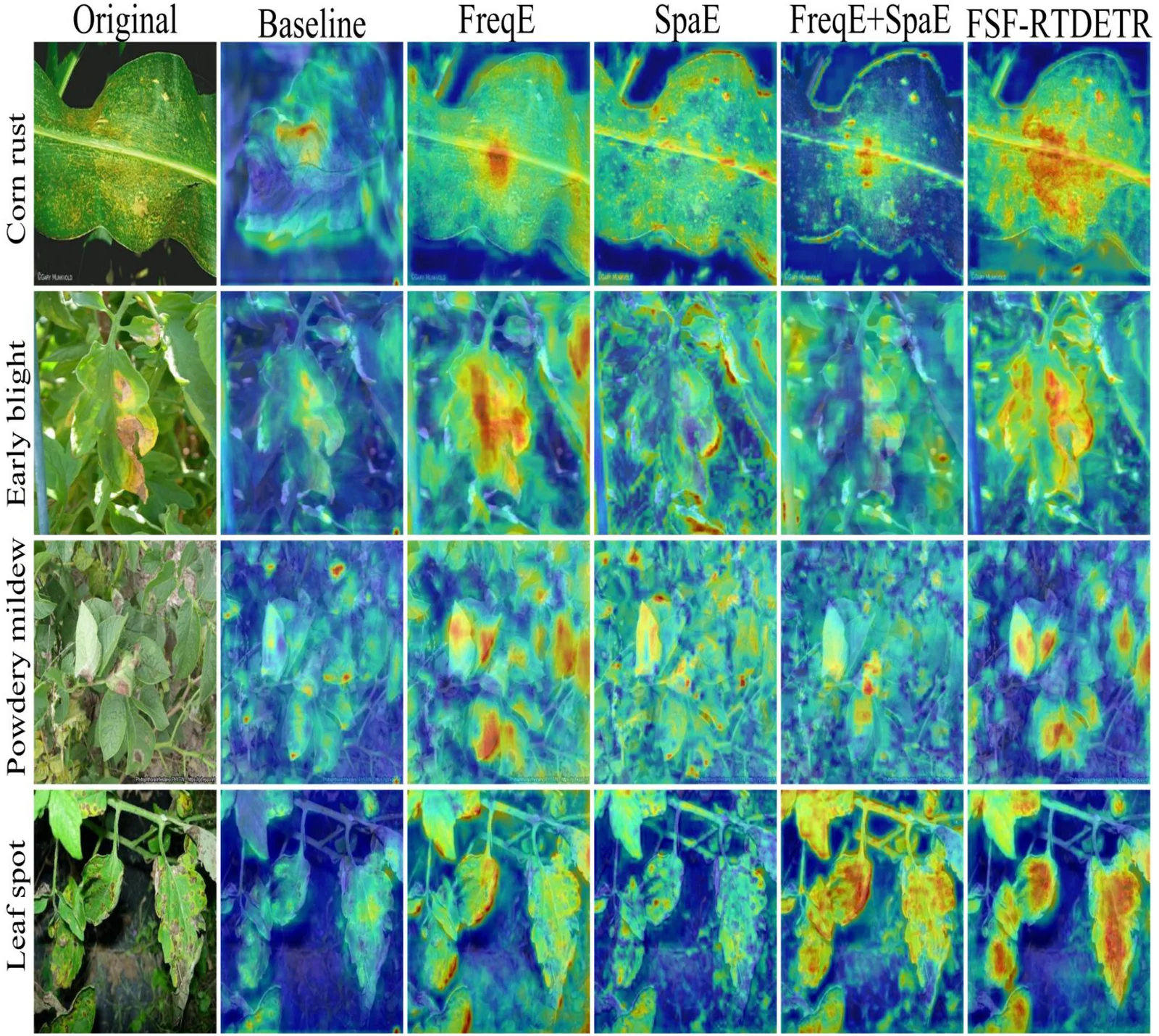
Grad-CAM of ablation modules in FSF-RTDETR on the PlantDoc dataset.

As shown in **Table 6**, SpaE, FreqE, and EGMuF improve the model performance to varying degrees. After introducing SpaE, the Recall increases from 53.1% to 57.8%, indicating that the spatial enhancement branch helps capture the contextual relationships between diseased regions and the surrounding leaf background, thereby reducing missed detections. With the introduction of FreqE, mAP_50_ and mAP_50:95_ increase to 61.2% and 49.7%, respectively, demonstrating that the frequency enhancement branch strengthens the representation of disease textures, edges, and fine-grained abnormal patterns in leaf images. When EGMuF is introduced alone, mAP_50_ and mAP_50:95_ reach 68.9% and 55.5%, respectively, showing a notable improvement. This suggests that frequency–spatial feature fusion enhances the model’s ability to discriminate among different disease morphologies. Further comparison of the module combinations shows that FreqE + SpaE, FreqE + EGMuF, and SpaE + EGMuF all outperform the baseline model, indicating the complementarity among the proposed modules. When FreqE, SpaE, and EGMuF are jointly integrated, the complete FSF-RTDETR achieves the highest mAP_50_ and mAP_50:95_ values among all experimental settings, reaching 70.8% and 58.2%, respectively. Although its FPS is 81.02, which is lower than that of the baseline model, it still maintains a relatively high inference speed. Overall, FSF-RTDETR achieves a favorable balance among detection accuracy, model complexity, and inference efficiency on the PlantDoc dataset, demonstrating the generalization capability of the proposed method in complex plant disease scenarios. **Figure 14** presents the detection performance of different ablation models on representative categories from the PlantDoc dataset. Overall, FSF-RTDETR shows relatively balanced performance across the four disease categories, indicating that FreqE, SpaE, and EGMuF have complementary effects. For corn rust, all models achieve generally good results, suggesting that rust-like lesions have relatively distinct visual characteristics and are easier to detect. For early blight and powdery mildew, the lesions usually show irregular shapes and weak boundaries, making detection more challenging. In these cases, FSF-RTDETR provides more stable performance in terms of mAP_50_ and mAP_50:95_. For tomato *Septoria* leaf spot, FSF-RTDETR shows certain advantages in Precision and mAP_50_, indicating its stronger discriminative ability for detecting leaf spot diseases. These results show that FSF-RTDETR maintains reliable detection performance across different disease morphologies, further verifying the effectiveness and generalization ability of the proposed modules on the PlantDoc dataset.

**Figure 14:**
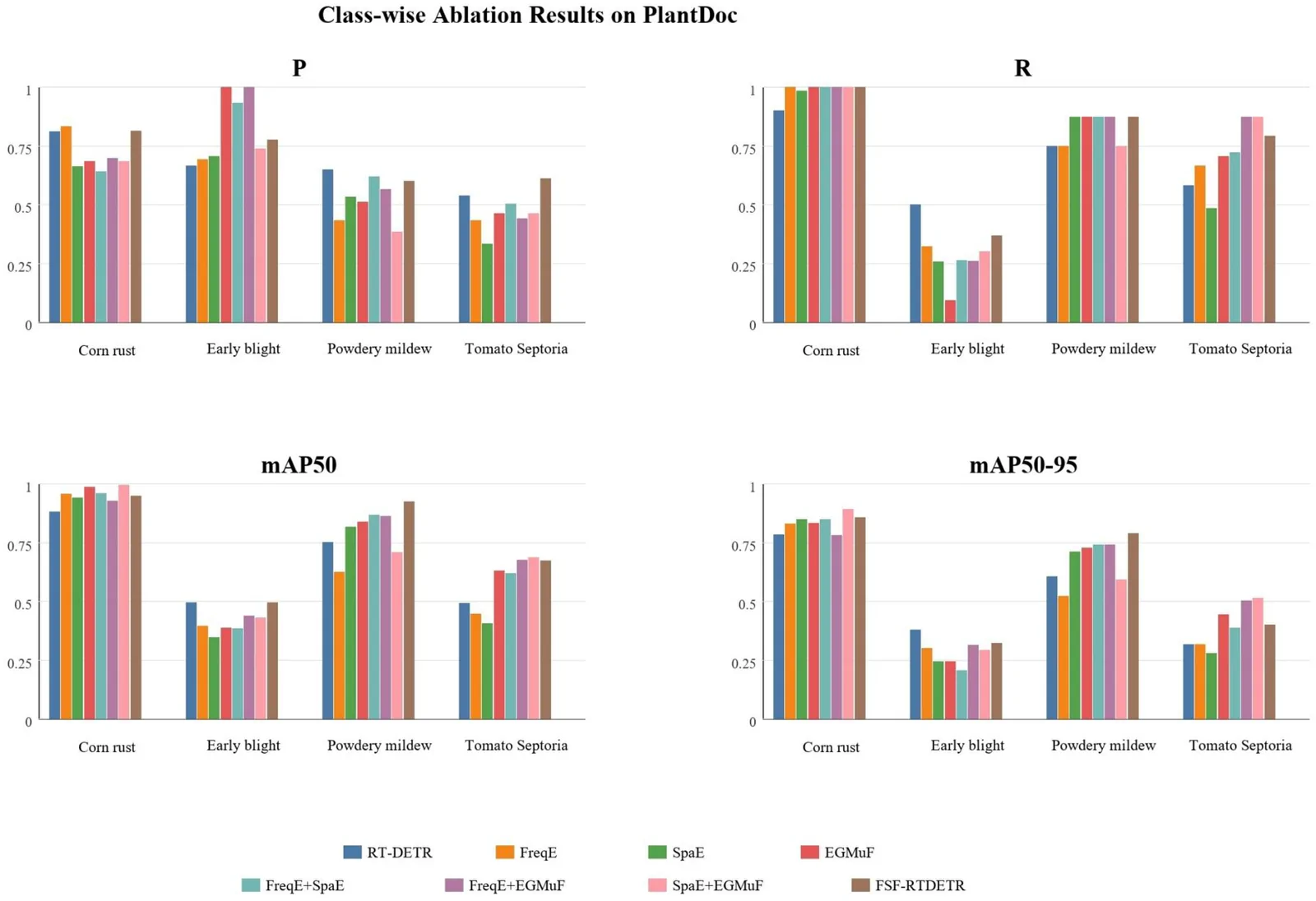
Performance of FSF-RTDETR ablation models on typical categories in the PlantDoc dataset.

##### 5.2.2.2 Visualization of ablation experiments

**Figure 13** shows the Grad-CAM heatmaps of different ablation models, illustrating each model’s attention to disease regions. **Figure 15** presents the corresponding detection results, enabling comparison of the localization and recognition performance of different module combinations in complex plant disease scenarios.

**Figure 15:**
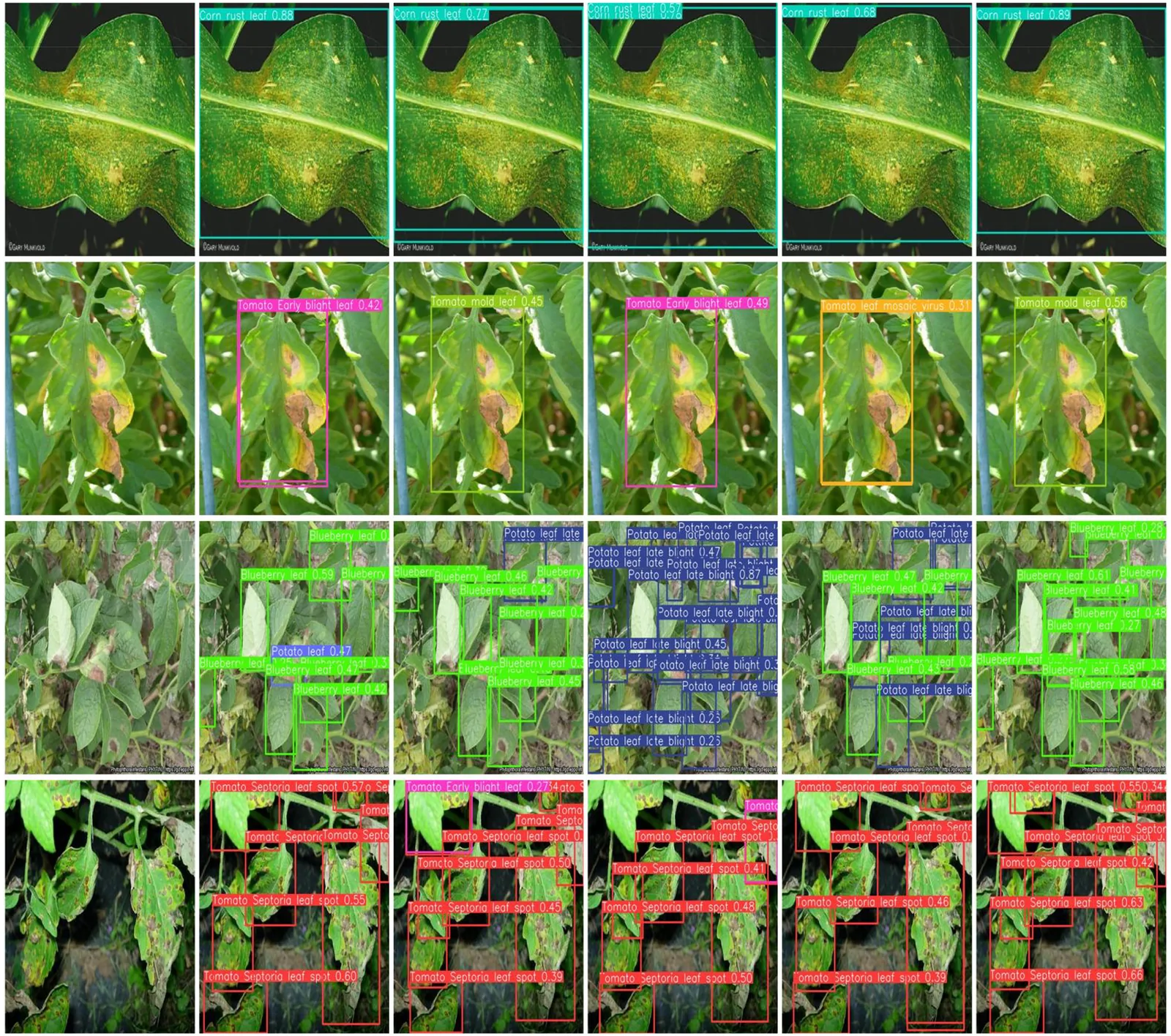
Visualization of detection results of different fusion modules on the PlantDoc dataset.

As shown in **Figure 13**, the proposed modules remain effective in cross-dataset scenarios. Compared with the baseline model, the frequency enhancement branch improves the recognition of fine-grained texture diseases such as corn rust and powdery mildew. This indicates that frequency-domain features help enhance local texture responses, such as rust-like spots and powdery disease patterns. After introducing the spatial enhancement branch, the model shows more stable performance on early blight and leaf spot, which often have larger lesion regions, irregular shapes, or complex backgrounds. This suggests that spatial dependency modeling helps the model better understand leaf structures, lesion distributions, and background relationships. With the further introduction of EGMuF, the class-wise performance becomes more balanced, indicating that frequency–spatial feature fusion can adaptively integrate effective information under different disease appearances. **Figure 15** shows that the baseline model tends to produce scattered attention, incomplete localization, or false detections in samples with complex backgrounds and large lesion-scale variations. After introducing FreqE, the model shows stronger responses to fine-grained texture regions, such as rust-like spots and powdery disease patterns. This indicates that frequency-domain features help enhance lesion edges and the representation of local textures. After adding SpaE, the model models the spatial relationships between leaf bodies and disease regions more stably, and its localization performance improves under occlusion or cluttered backgrounds. With the further introduction of EGMuF, the model can jointly utilize frequency-domain details and spatial semantic information. Its attention becomes more concentrated on the main disease regions, and the detection boxes are more complete. Overall, FSF-RTDETR shows more stable performance on typical samples such as corn rust, early blight, powdery mildew, and leaf spot, indicating that the proposed modules have good generalization across the PlantDoc dataset.

### 5.3 Comparative experiments

To further evaluate the detection performance of the proposed FSF-RTDETR on the Eggplant dataset and PlantDoc dataset, this study selects 19 mainstream object detection models for comparison. The comparison models include YOLO-series models, such as YOLOv8m, YOLO10M, YOLO11M, YOLO12M, YOLO13L, and YOLO26M; improved YOLO/Mamba-based models, such as YOLO-master-s, Mamba-YOLO-B, hyper-yolo-M, and MHAF-YOLO-m; and other types of detectors, including Faster R-CNN, RetinaNet, Task-Aligned One-Stage Object Detection (TOOD), Deformable DETR, DETR with Improved Matching for Fast Convergence (DEIM)-series models, and RT-DETR-R18. These models cover one-stage detectors, two-stage detectors, and end-to-end Transformer-based detectors, enabling a comprehensive comparison of the proposed method in different crop disease and pest detection scenarios. The main evaluation metrics include Precision, Recall, mAP_50_, mAP_50:95_, Parameters, and GFLOPs.

#### 5.3.1 Comparative experiments on the Eggplant dataset

**Table 6** presents the quantitative comparison results of different models on the Eggplant dataset. To further examine the practical detection performance of different models, four representative classes, namely FruitRot, FruitBorer, Healthy, and MelonThrips’s P, are selected for visual comparison, as shown in **Figures 16**–**19**. Specifically, **Table 7** summarizes the precision results for these four classes: FruitRot (FR) is used to observe the localization performance for large rot lesions; FruitBorer (FB) is used to analyze the ability of the models to detect small borer holes; MelonThrips (MT) is used to compare the recognition ability for fine-textured bite marks; and Healthy (HT) is used to evaluate false detections in healthy fruit scenes.

**Figure 16:**
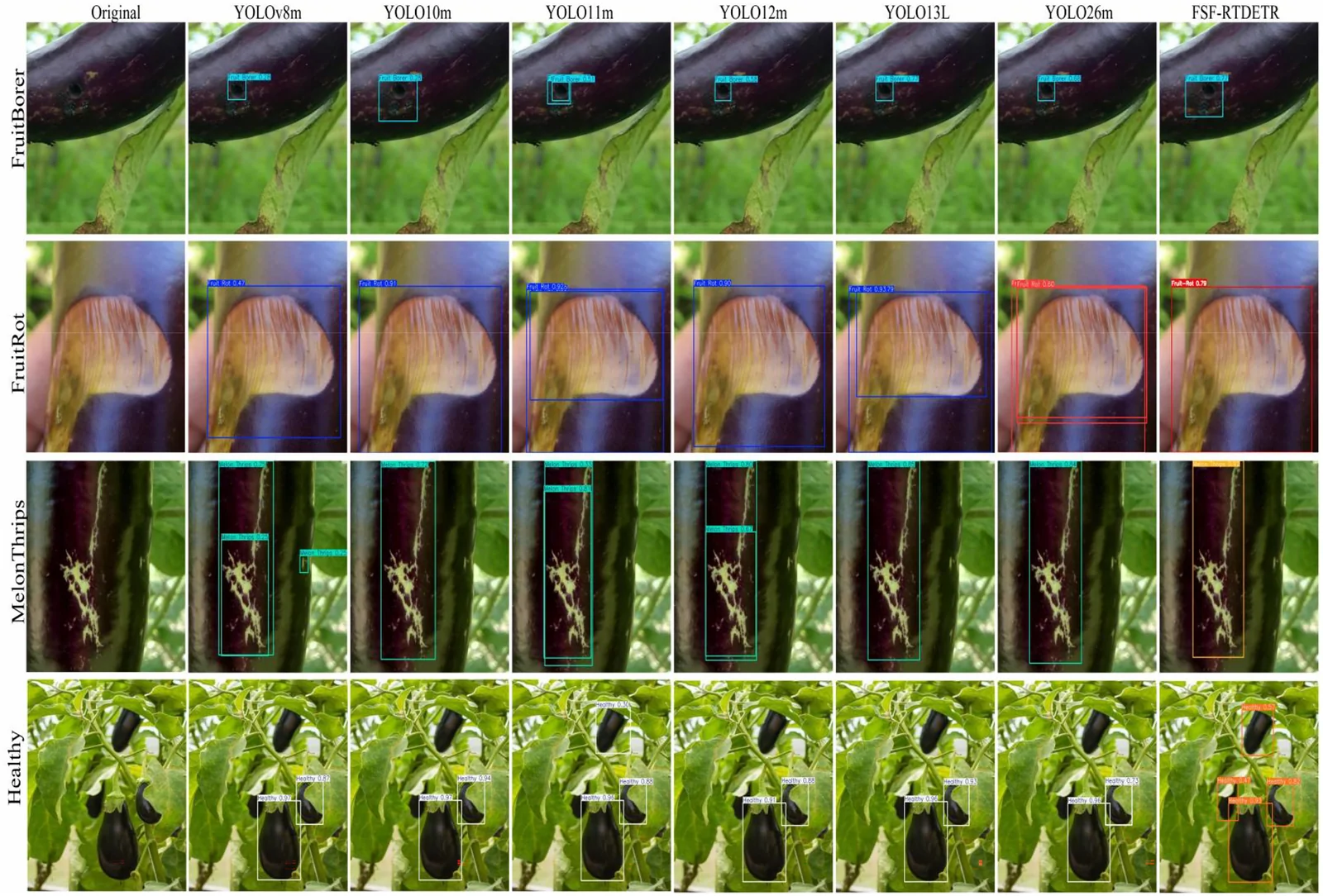
Visualization of YOLO serial modules on the eggplant dataset.

**Figure 17:**
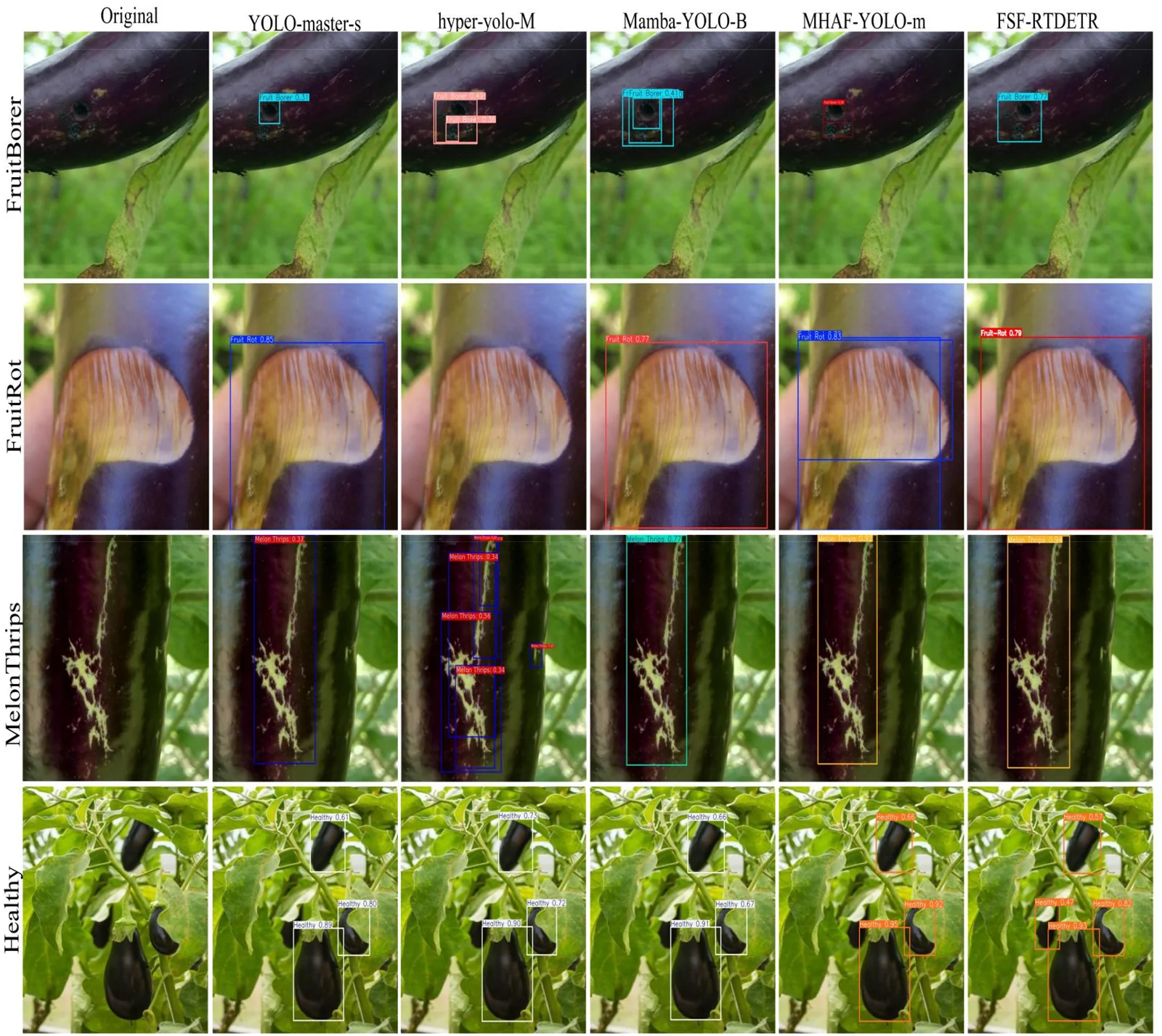
Visualization comparison of improved YOLO/Mamba-based models on the eggplant dataset.

**Figure 18:**
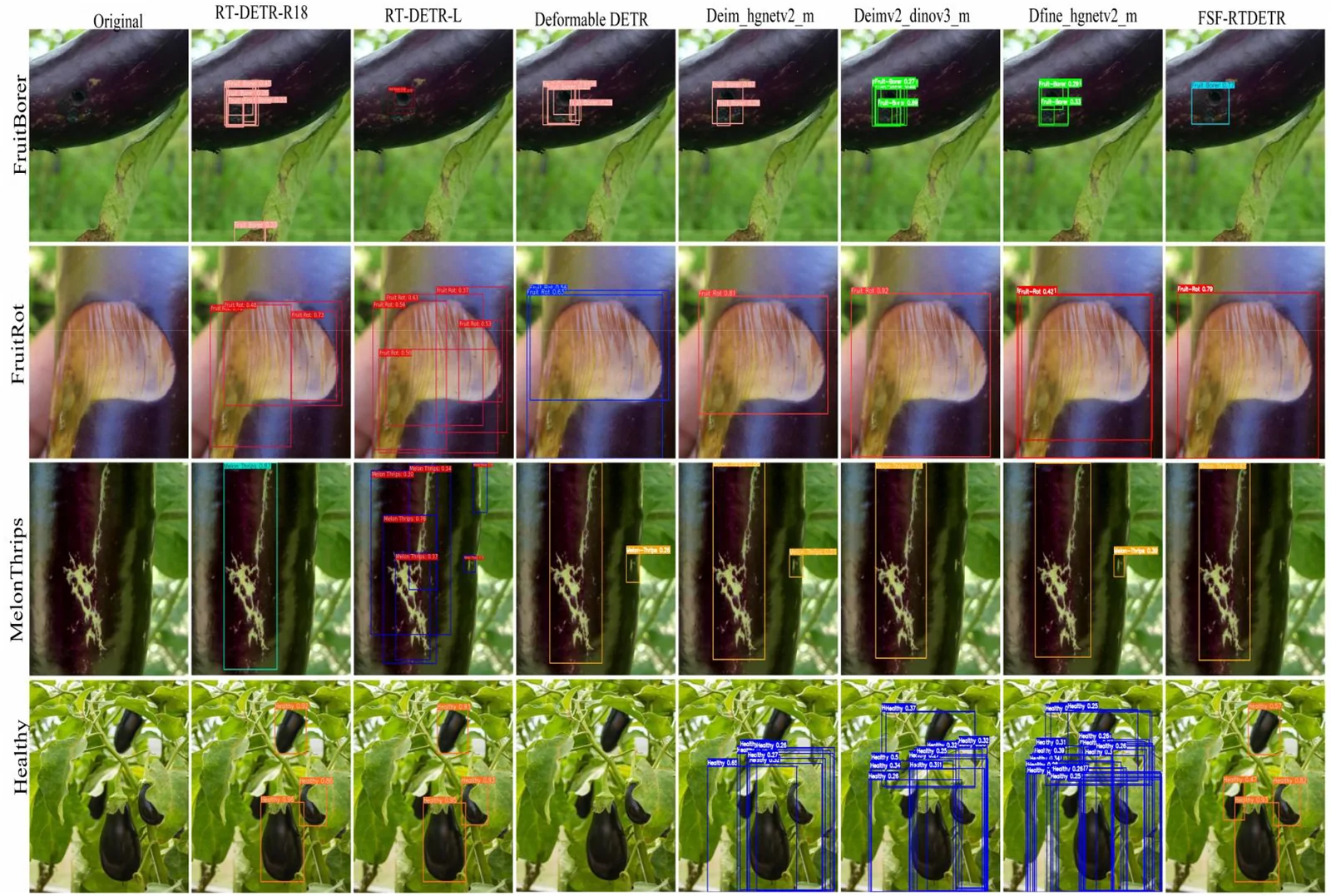
Visualization of DETR serial modules on the eggplant dataset.

**Figure 19:**
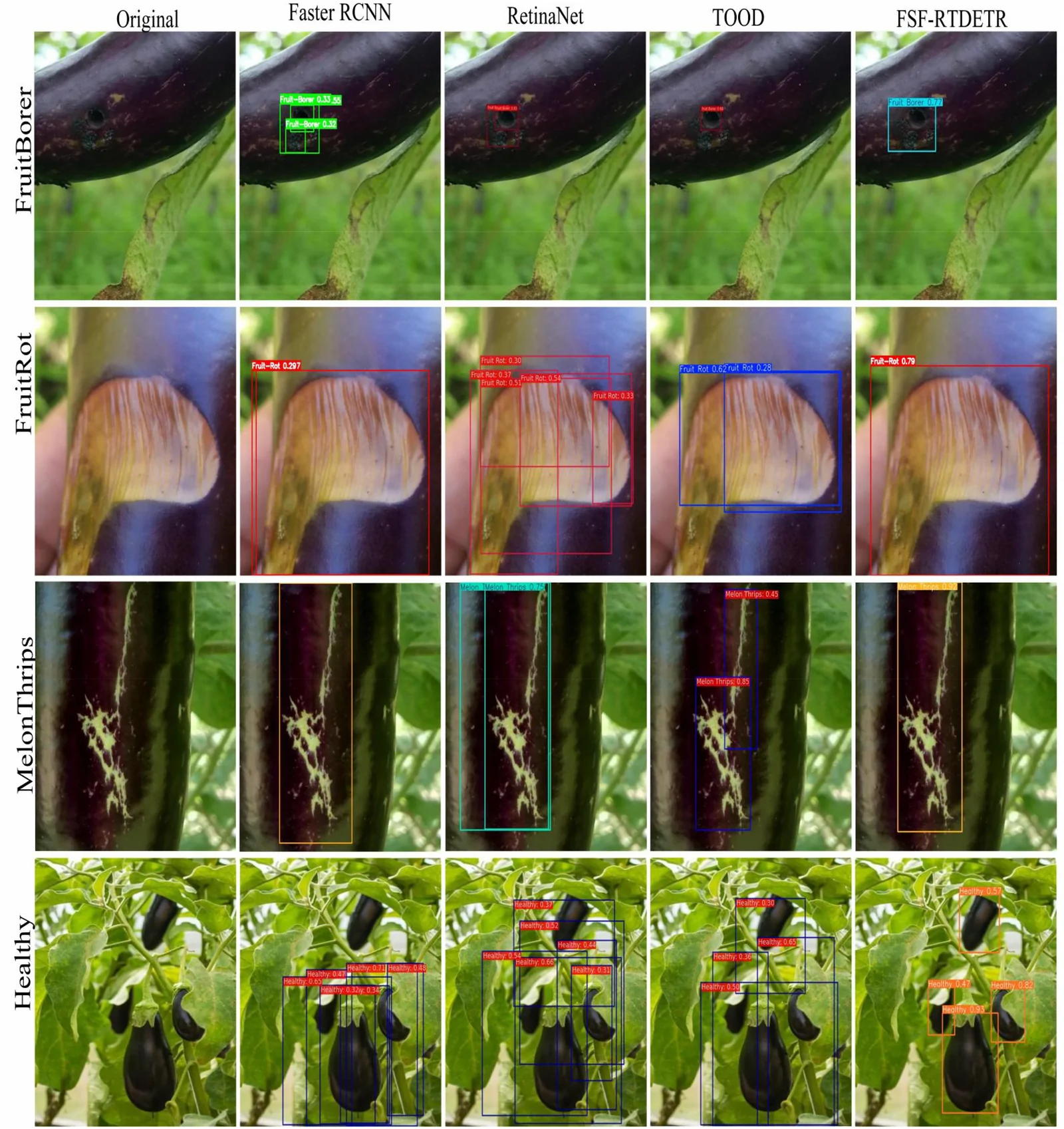
Visualization of traditional detection models on the eggplant dataset.

**Table 7:** Class-wise precision and performance comparison of different models on the Eggplant dataset.

Compared models	FR	FB	HT	MT	*p*-value (%)	*R* (%)	mAP_50_ (%)	mAP5_0:95_ (%)	Params	GFLOPs	FPS
YOLOv8m (Yaseen, 2024)	79.5	76.5	89.6	55.2	73.9	68.2	74.2	45.7	25.85M	79.1G	128.21
YOLO10M (Wang et al., 2024a)	73.6	75.5	88.2	69	76.6	69.5	72.3	44.7	16.48M	64.0G	158.73
YOLO11M (Khanam and Hussain, 2024)	77	73.3	86.5	68.7	76.4	68.4	73.2	45.7	20.05M	68.2G	117.65
YOLO12M (Tian et al., 2026)	80.3	75.5	86.8	68.2	77.7	69.5	73.9	45.8	20.14M	67.8G	116.28
YOLO13L (Lei et al., 2025)	75.4	72.2	90.9	66.3	76.2	71.9	74.3	48.9	27.58M	89.1G	82.64
YOLO26M (Sapkota et al., 2025)	77.5	73.6	86.4	61.8	74.8	69.5	74.0	45.1	20.35M	67.9G	196.08
YOLO-master-s (Lin et al., 2025)	78.4	76.7	88.8	69.3	78.3	69.2	73.4	46.3	29.13M	34.7G	270.27
Mamba-YOLO-B (Wang et al., 2025b)	76.1	75.9	90.6	70.2	78.2	69.5	73.5	46.8	20.51M	44.6G	96.15
Hyper-YOLO-M (Feng et al., 2025)	75.1	76.7	84.8	61	74.4	70.8	73.9	46.2	30.73M	92.6G	105.26
MHAF-YOLO-m (Yang et al., 2025)	80	75.1	89.4	63.3	77.0	67.0	71.5	43.9	16.60M	71.5G	148.68
DEIM-HGNetv2-M (Huang et al., 2025b)	76.8	82.8	21.9	40.6	61.5	57.5	55.5	27.4	19.21M	56.45G	141.57
DEIMv2-DINOv3-M (Huang et al., 2025a)	76.4	85.3	41.3	53.7	64.2	59.5	60.0	30.3	18.07M	51.98G	147.63
DFINE-HGNetv2-M (Peng et al., 2025)	66.3	79.6	29.2	47.3	55.6	58.0	56.0	28.0	19.21M	56.45G	141.57
RT-DETR-L (Zhao et al., 2024)	81.7	80.5	87.2	69.6	79.7	71.5	74.7	46.9	31.99M	103.4G	48.29
RetinaNet (Lin et al., 2020)	71.7	81.1	35.7	46.1	58.7	54.0	53.2	22.3	36.93M	139.26G	78.30
TOOD (Feng et al., 2021)	73.6	82.1	36.9	43.4	59.0	55.5	55.1	23.8	32.09M	126.98G	51.60
Faster R-CNN (Ren et al., 2017)	78.8	82.6	30.9	54.1	61.6	53.4	54.9	23.8	41.49M	137.21G	70.40
Deformable DETR (Zhu et al., 2020)	63.7	72.7	35.1	39.8	52.8	49.1	47.9	20.6	39.82M	204.91G	44.70
Baseline (Zhao et al., 2024)	81.3	82.5	91.7	69.4	79.8	69.3	74.6	46.0	19.87M	57.0G	227.27
FSF-RTDETR (OURS)	**81.6**	**83.8**	**93.3**	**79.7**	**81.5**	**71.3**	**78.8**	**50.7**	**21.54M**	**51.7G**	**104.07**

Bold values indicate the experimental results of the proposed model.

As shown in **Table 7**, FSF-RTDETR (OURS) performs well across all four categories. It achieves the best results on Healthy and MelonTrips, reaching 93.3% and 79.7%, respectively. The improvement on MelonTrips is particularly notable, exceeding that of the best competing model by 9.5 percentage points. For FruitRot, its result of 81.6% is only 0.1 percentage points below that of RT-DETR-L. It also obtains 83.8% on FruitBorer, ranking second among the compared models. The average score across the four categories is 84.6%, which is 3.4 percentage points higher than the baseline. These results show that FSF-RTDETR improves the detection of difficult pest categories while maintaining stable performance on the other classes. Moreover, FSF-RTDETR achieves superior overall detection performance on the eggplant fruit pest and disease dataset. Compared with the other detection models, the proposed model obtains the highest Precision, mAP_50_, and mAP_50:95_ values, reaching 81.5%, 78.8%, and 50.7%, respectively. This indicates that FSF-RTDETR has advantages in pest and disease target recognition and localization accuracy. Compared with the baseline RT-DETR-R18, the mAP_50_ of FSF-RTDETR increases from 74.6% to 78.8%, while the mAP_50:95_ increases from 46.0% to 50.7%, corresponding to improvements of 4.2 and 4.7 percentage points, respectively. These results demonstrate that the frequency enhancement, spatial modeling, and frequency–spatial fusion mechanisms effectively improve the detection of weak small lesions, fine-grained texture patterns, and targets under complex backgrounds. In terms of model complexity, FSF-RTDETR has 21.54M parameters and 51.7 GFLOPs, which are lower than the corresponding values for RT-DETR-L, YOLO13L, hyper-yolo-M, Faster R-CNN, and Deformable DETR. Although its FPS is 104.07, which is lower than that of some lightweight YOLO models, it still satisfies the requirements of practical detection. Overall, FSF-RTDETR achieves a favorable balance between detection accuracy and model complexity on the eggplant dataset.

**Figures 16**-**19** show clear performance differences among the compared models in complex eggplant scenarios. FSF-RTDETR provides more accurate localization for tiny FruitBorer lesions and more stable coverage of large, irregular FruitRot regions. For MelonThrips damage, the proposed model reduces background misclassification caused by weak textures and fruit surface interference. In Healthy samples with occluded and overlapping fruits, FSF-RTDETR also produces fewer false detections. These results indicate that the proposed method is more robust for detecting small targets, weak-texture diseases, and targets under complex backgrounds.

#### 5.3.2 Comparative experiments on the PlantDoc dataset

To validate the generalization and complex disease adaptability of FSF-RTDETR, comparative experiments are further carried out on the PlantDoc dataset. **Table 8** shows the quantitative evaluation metrics on the PlantDoc dataset. **Figures 20**–**23** present the P(precision) detection comparisons of different models on four typical diseases: corn rust (CR), powdery mildew (PM), early blight (EB), and tomato *Septoria* leaf spot (TSLS). Corn rust is used to test the model’s performance on dense rust-spot diseases, powdery mildew to assess weak-boundary powdery disease recognition, early blight to evaluate large irregular lesion localization, and tomato *Septoria* leaf spot to assess detection under complex leaf-spot backgrounds.

**Table 8:** Class-wise precision and performance comparison of different models on the PlantDoc dataset.

Compared models	CR	PM	EB	TSLS	p-value (%)	R (%)	mAP_50_ (%)	mAP_50:95_ (%)	Params	GFLOPs	FPS
YOLOv8M (Yaseen, 2024)	85.3	82.5	35.6	51.8	60.3	64.9	66.2	52.2	25.85M	79.1G	109.89
YOLO10M (Wang et al., 2024a)	86.7	72.9	44.1	50.8	56.8	65.2	64.1	51.0	16.48M	64.0G	103.09
YOLO11M (Khanam and Hussain, 2024)	85.3	55.4	77.8	43.0	60.1	61.6	63.9	49.6	20.05M	68.2G	96.15
YOLO12M (Tian et al., 2026)	81.5	67.5	49.6	49.4	57.3	66.4	65.6	51.7	20.14M	67.8G	87.72
YOLO13L (Lei et al., 2025)	53.6	44.5	44.2	58.7	55.0	52.7	58.9	48.0	27.58M	89.1G	64.52
YOLO26M (Sapkota et al., 2025)	85.6	58.9	33.8	59.0	59.5	58.3	64.1	48.9	20.35M	67.9G	131.58
YOLO-master-s (Lin et al., 2025)	82.9	65.4	44.6	40.7	55.9	55.4	58.4	45.6	29.13M	34.7G	200.00
Mamba-YOLO-B (Wang et al., 2025b)	83.4	65.1	54.7	54.3	61.5	60.9	65.9	51.8	20.51M	44.6G	89.29
Hyper-YOLO-M (Feng et al., 2025)	86.0	70.6	45.3	59.1	56.6	64.0	65.8	53.3	30.73M	92.6G	103.09
MHAF-YOLO-M (Yang et al., 2025)	78.5	66.8	41.2	43.5	54.8	60.8	61.7	48.5	16.60M	71.5G	149.36
DEIM-HGNetv2-M (Huang et al., 2025b)	81.0	64.1	39.3	27.4	61.1	66.2	66.8	53.5	19.21M	56.45G	138.92
DEIMv2-DINOv3-M (Huang et al., 2025a)	87.8	70.8	45.9	43.1	63.3	67.1	67.3	52.9	18.07M	51.98G	146.31
DFINE-HGNetv2-M (Peng et al., 2025)	82.3	60.4	65.3	47.3	61.2	69.8	69.4	55.9	19.21M	56.45G	138.92
RT-DETR-L (Zhao et al., 2024)	68.0	63.3	55.3	52.4	60.1	60.2	58.0	44.8	32.04M	103.4G	36.23
RetinaNet (Lin et al., 2020)	25.3	41.0	10.5	12.9	34.2	43.6	32.7	32.8	36.93M	139.26G	74.60
TOOD (Feng et al., 2021)	30.8	89.0	90.0	30.6	53.6	36.4	37.6	24.4	32.09M	126.98G	48.30
Faster R-CNN (Ren et al., 2017)	38.3	33.5	28.2	38.7	32.4	50.7	44.2	30.8	41.49M	137.21G	69.50
Deformable DETR (Zhu et al., 2020)	47.1	80.0	20.8	19.5	41.7	51.7	46.1	34.4	39.82M	204.91G	46.10
Baseline (Zhao et al., 2024)	81.7	67.2	65.4	55.1	66.5	53.1	53.7	42.8	19.87M	57.0G	101.01
FSF-RTDETR (OURS)	**87.3**	**78.6**	**61.8**	**62.5**	**68.2**	**69.2**	**70.8**	**58.2**	**21.54M**	**51.7G**	**81.02**

Bold values indicate the experimental results of the proposed model.

**Figure 20:**
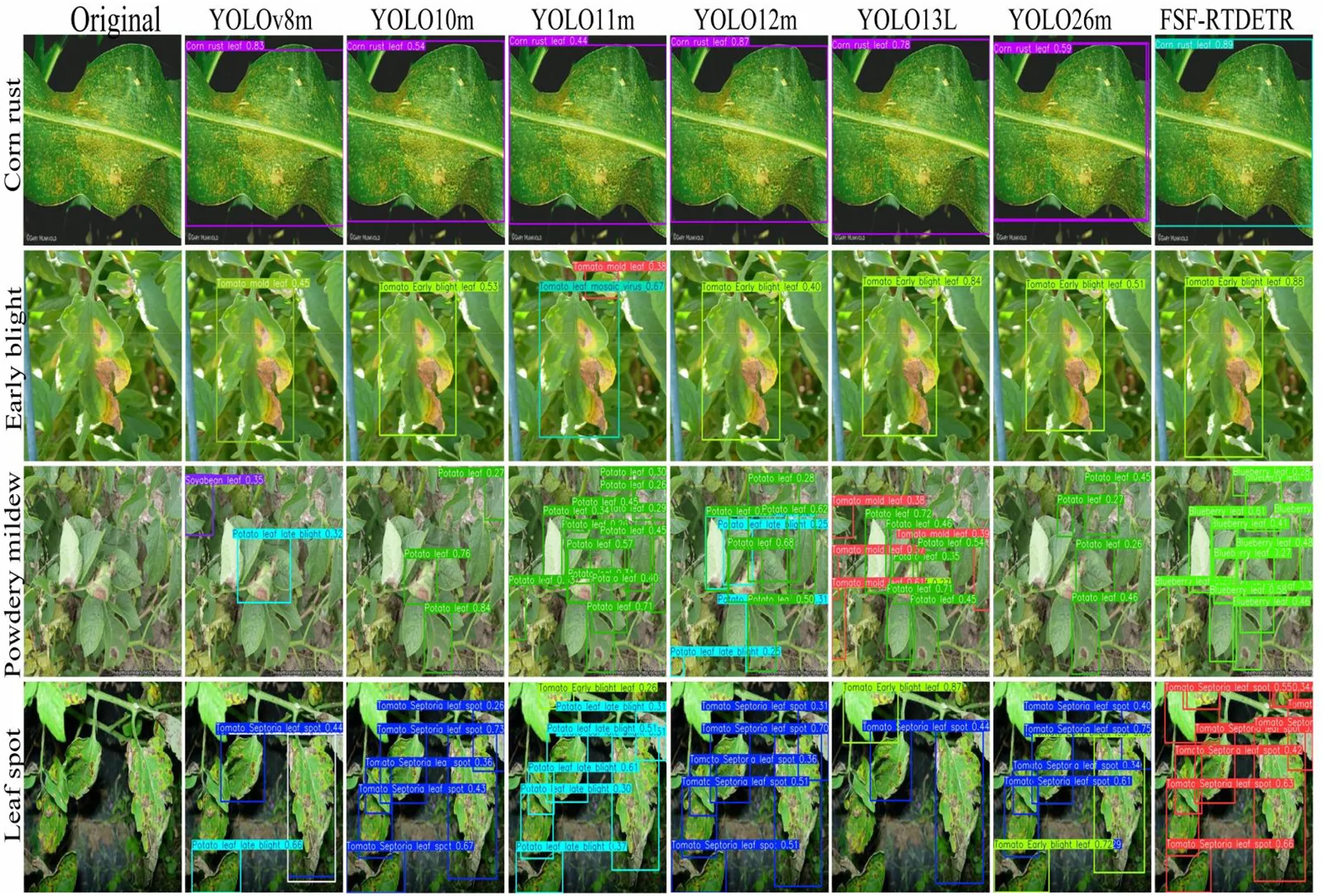
Visualization of YOLO serial modules on the PlantDoc dataset.

**Figure 21:**
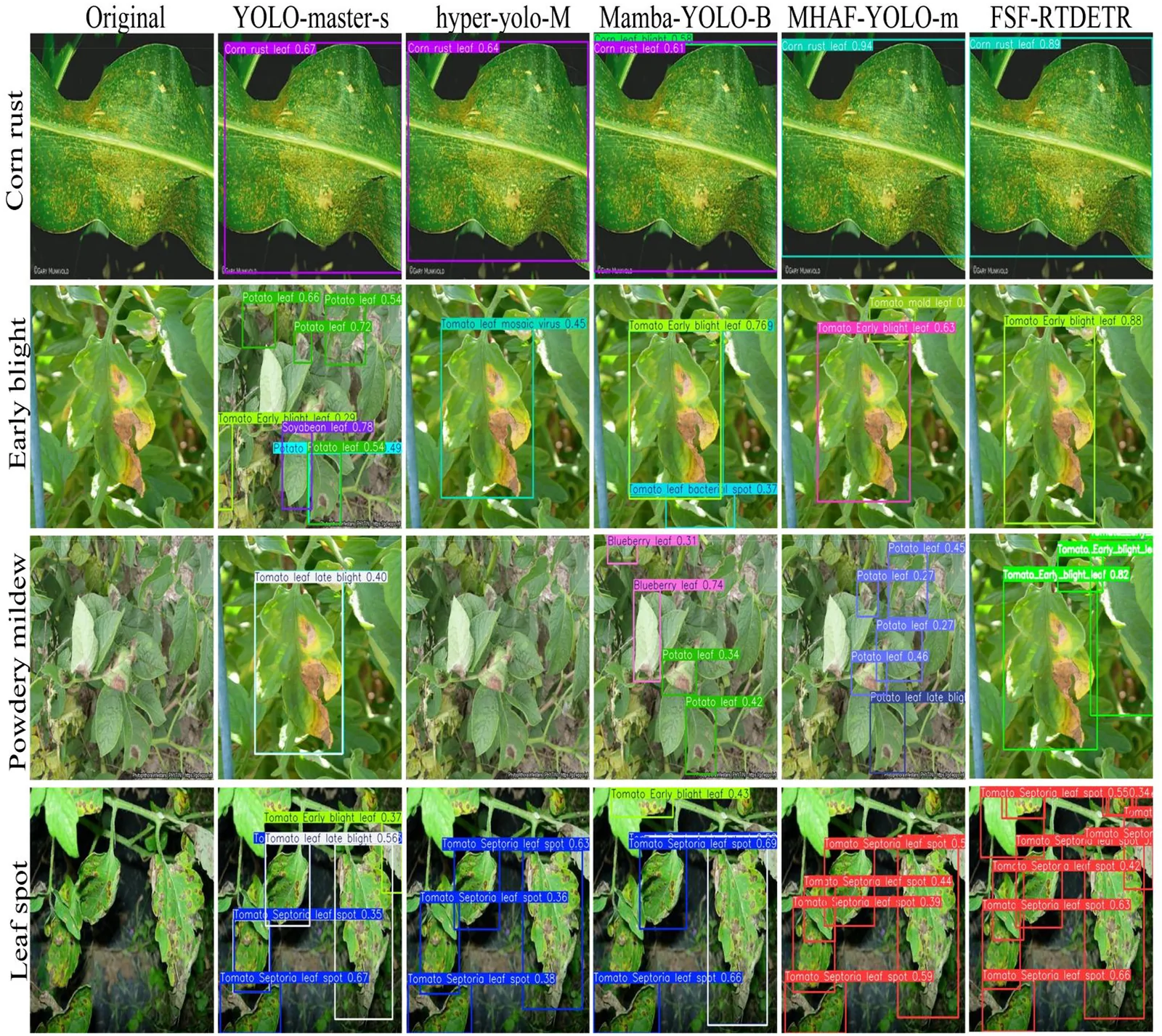
Visualization comparison of improved YOLO/Mamba-based models on the PlantDoc dataset.

**Figure 22:**
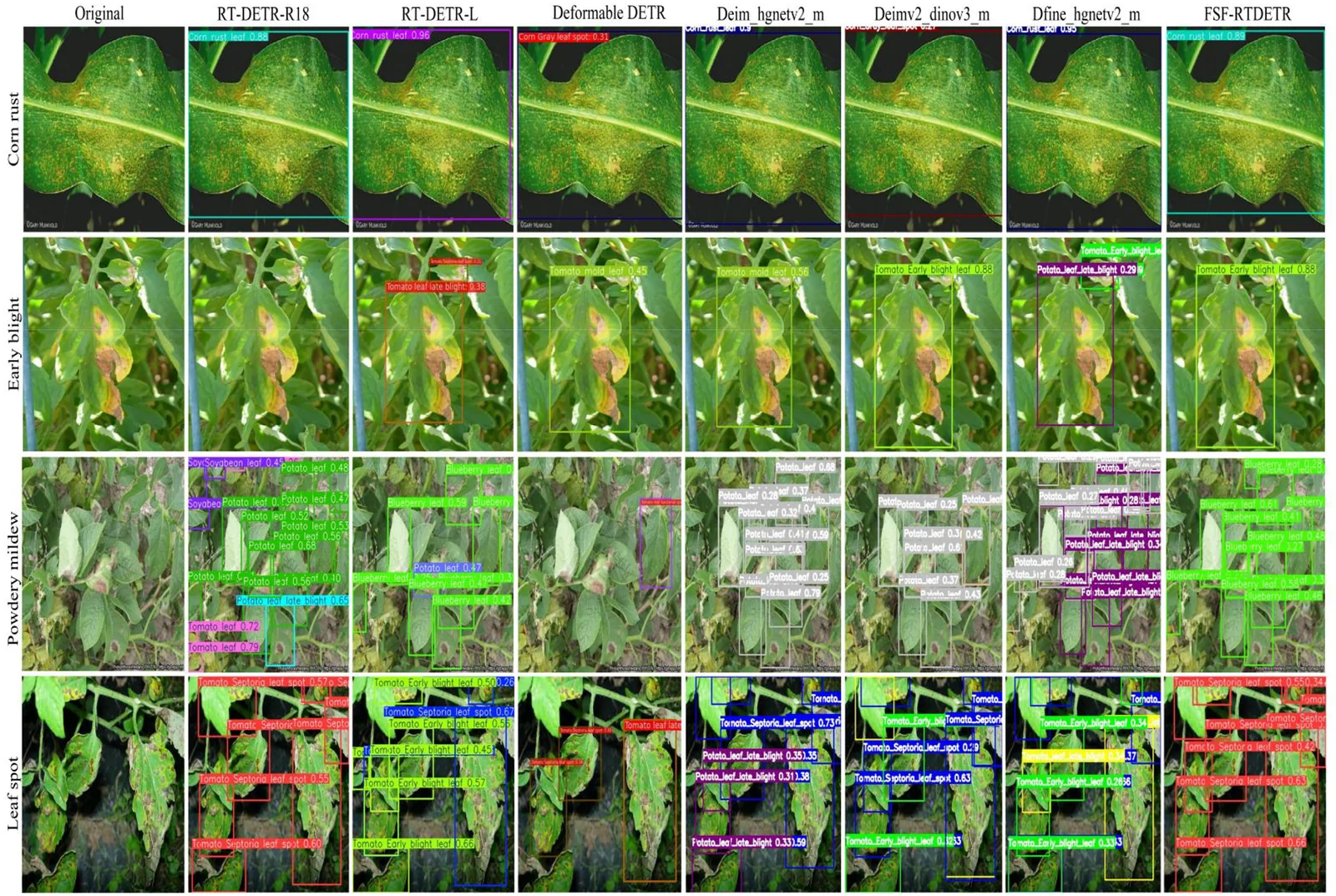
Visualization of DETR serial modules on the PlantDoc dataset.

**Figure 23:**
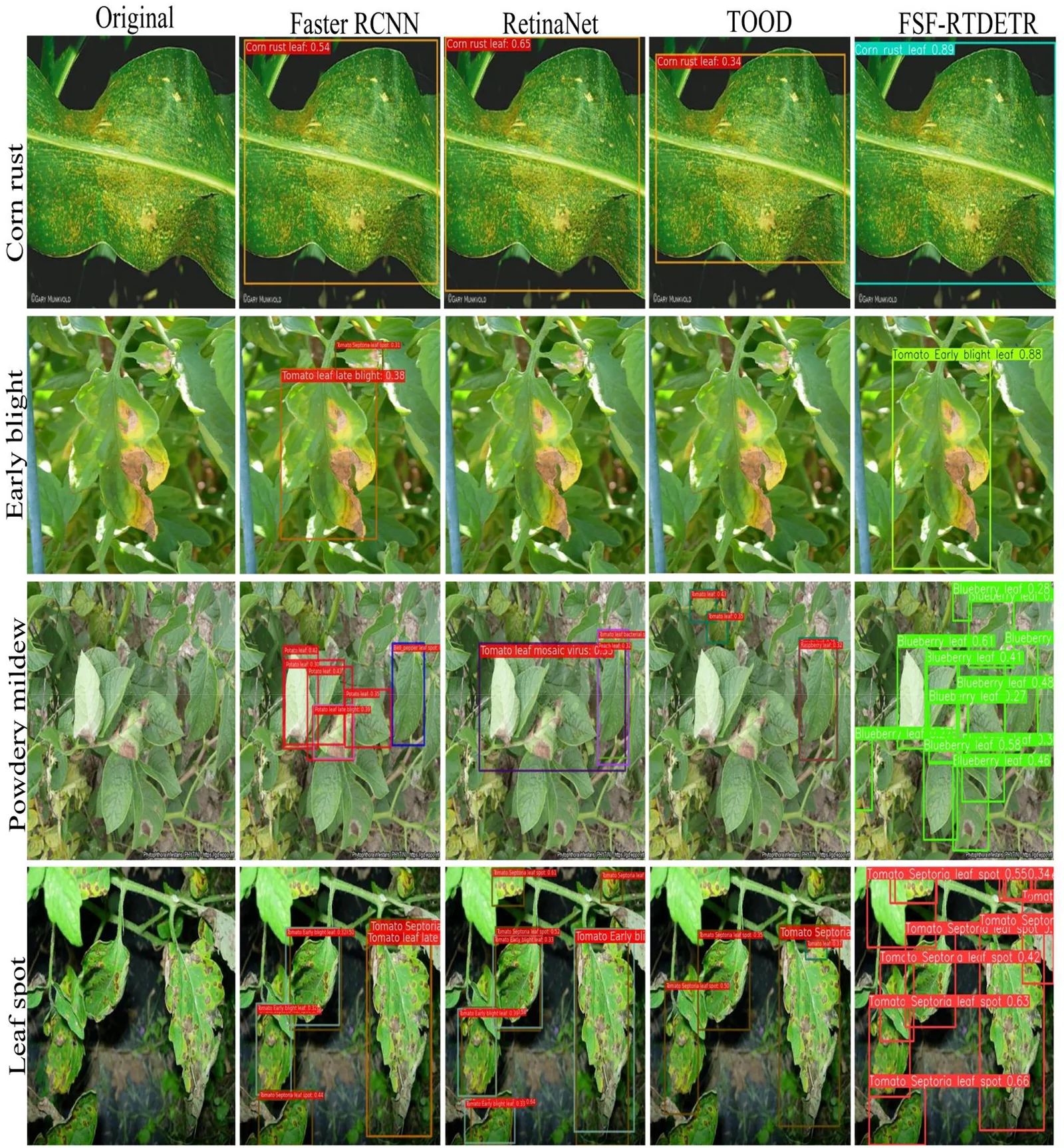
Visualization of traditional detection models on the PlantDoc dataset.

**Table 8** compares the class-wise results for four representative diseases: CR, PM, EB, and TSLS. FSF-RTDETR achieves a score of 87.3% for CR and the highest result of 62.5% on TSLS. Its performance on PM reaches 78.6%, while the result for EB is 61.8%. Compared with the baseline, the proposed model improves CR, PM, and TSLS by 5.6, 11.4, and 7.4 percentage points, respectively, although EB decreases by 3.6 percentage points. Across the four categories, FSF-RTDETR obtains an average score of 72.6%, exceeding the baseline by 5.2 percentage points. The results indicate that the proposed model provides a more balanced performance across diseases with different visual characteristics. FSF-RTDETR achieves superior overall detection performance on the PlantDoc dataset. Compared with the YOLO-series models, FSF-RTDETR shows a more pronounced advantage in mAP_50:95_, indicating more stable localization performance for lesion boundaries and multiscale targets. Compared with Deformable DETR, Faster R-CNN, RetinaNet, and TOOD, the proposed method achieves substantial accuracy improvements while maintaining a relatively low parameter count and computational complexity. Although the FPS of FSF-RTDETR is lower than that of several lightweight YOLO, DEIM, and DFINE models, it still reaches 81.02 FPS, which satisfies the real-time detection requirements. Overall, FSF-RTDETR achieves a favorable balance among detection accuracy, model scale, and inference speed on the PlantDoc dataset, outperforming most of the compared models. These results further validate the effectiveness of the proposed method in complex plant disease detection tasks.

**Figures 20**-**23** show clear performance differences among different models in complex natural scenarios of the PlantDoc dataset. Traditional detectors tend to suffer from missed detections, localization shifts, and category confusion under leaf occlusion, cluttered backgrounds, and blurred lesion boundaries. The YOLO-series models show high inference speed, but redundant boxes and unstable confidence scores can still occur when detecting small and multispot diseases. DETR-based variants benefit from global modeling, yet they can be affected by leaf textures and illumination variations when localizing fine-grained lesions. By contrast, FSF-RTDETR better covers diseased regions such as Corn rust and Early blight, with more consistent bounding boxes and confidence scores. It also detects more lesion regions in dense-spot and heavily overlapped leaf scenarios. Nevertheless, minor duplicate detections and category confusion still exist for extremely small lesions, which may be further improved through finer annotations and more targeted data augmentation. These results indicate that the proposed method provides more stable detection performance under complex backgrounds, scale variations, and dense lesion distributions, thereby further supporting the effectiveness of the proposed improvements.

## 6 Conclusion

This study proposes FSF-RTDETR for eggplant fruit pest and disease detection under complex natural scenarios. The method is built on RT-DETR-R18 and introduces a dual-stream frequency–spatial enhanced backbone. By jointly modeling frequency-domain details and spatial contextual information, FSF-RTDETR improves the representation of small lesions, weak textures, occluded targets, and complex backgrounds. Specifically, the frequency enhancement branch strengthens lesion edges, high-frequency textures, and local abnormal responses, while the spatial enhancement branch improves the modeling of fruit contours, occlusion relationships, and background context. In addition, the EGMuF module adaptively integrates frequency-domain detailed features and spatial semantic features, allowing the model to obtain more discriminative representations for different pest and disease patterns. Experimental results show that FSF-RTDETR achieves Precision, Recall, mAP_50_, and mAP_50:95_ values of 81.5%, 71.3%, 78.8%, and 50.7%, respectively, on the Eggplant dataset. Compared with the baseline RT-DETR-R18, these results correspond to improvements of 1.7, 2.0, 4.2, and 4.7 percentage points. On the PlantDoc dataset, FSF-RTDETR improves mAP_50_ and mAP_50:95_ by 17.1 and 15.4 percentage points over the baseline, respectively. These results indicate that the proposed method is effective for eggplant fruit pest and disease detection and shows promising generalization ability in cross-dataset plant disease detection.

Nevertheless, the proposed method still has some limitations. The introduction of frequency-domain modeling, multibranch fusion, and Mamba-based spatial modeling increases the computational burden to some extent, resulting in lower inference speed than some lightweight YOLO models. In addition, false detections, missed detections, or category confusion may still occur in cases involving extremely small lesions, strong light reflection, or highly similar disease and background textures. Future work will focus on reducing the inference cost of the model and improving its robustness by incorporating finer lesion-level annotations and more diverse complex-scenario data augmentation, thereby enhancing its applicability in real field environments.

## Data Availability

The raw data supporting the conclusions of this article will be made available by the authors, without undue reservation.

## References

[B1] Ashok S. Kishore G. Rajesh V. Suchitra S. Sophia S. G. G. Pavithra B. (2020). “ Tomato leaf disease detection using deep learning techniques”, in: 2020 5th International Conference on Communication and Electronics Systems (ICCES) ( IEEE), 979–983. doi: 10.1109/ICCES48766.2020.9137986

[B2] Aslan M. F. Aslan B. Sabanci K. (2026). Frequency-domain vision transformers: Architectures, applications, and open challenges. Appl. Sci. 16, 2024. doi: 10.3390/app1604202430654563

[B3] Atila Ü. Uçar M. Akyol K. Uçar E. (2021). Plant leaf disease classification using EfficientNet deep learning model. Ecol. Inf. 61, 101182. doi: 10.1016/j.ecoinf.2020.10118242574925

[B4] Bari B. S. Islam M. N. Rashid M. Hasan M. J. Razman M. A. M. Musa R. M. . (2021). A real-time approach of diagnosing rice leaf disease using deep learning-based faster r-CNN framework. PeerJ Comput. Sci. 7, e432. doi: 10.7717/peerj-cs.432 33954231 PMC8049121

[B5] Bohol Island State University (2023). Eggplant Disease Detection Dataset. Available online at: https://universe.roboflow.com/bohol-island-state-university-vgjlb/eggplant-disease-detection (Accessed October 17, 2026).

[B6] Carion N. Massa F. Synnaeve G. Usunier N. Kirillov A. Zagoruyko S. (2020). “ End-to-end object detection with transformers”, in: European conference on computer vision ( Springer), 213–229. doi: 10.48550/arXiv.2005.12872

[B7] Chen L. Gu L. Li L. Yan C. Fu Y. (2025). “ Frequency dynamic convolution for dense image prediction”, in: Proceedings of the IEEE/CVF conference on computer vision and pattern recognition, Piscataway, NJ: IEEE, 30178–30188. doi: 10.48550/arXiv.2503.18783

[B8] Chi L. Jiang B. Mu Y. (2020). Fast fourier convolution. Adv. Neural Inf. Process. Syst. 33, 4479–4488. Available online at: https://proceedings.neurips.cc/paper_files/paper/2020/hash/2fd5d41ec6cfab47e32164d5624269b1-Abstract.html.

[B9] Dong Q. Han T. Wu G. Sun L. Lu Y. (2026). Robust object detection for UAVs in foggy environments with spatial-edge fusion and dynamic task alignment. Remote Sens. 18, 169. doi: 10.3390/rs1801016930654563

[B10] Feng C. Zhong Y. Gao Y. Scott M. R. Huang W. (2021). “ TOOD: Task-aligned one-stage object detection”, in: Proceedings of the IEEE/CVF international conference on computer vision, 3490–3499. doi: 10.1109/ICCV48922.2021.00349

[B11] Feng Y. Huang J. Du S. Ying S. Yong J.-H. Li Y. . (2025). Hyper-YOLO: When visual object detection meets hypergraph computation. IEEE Trans. Pattern Anal. Mach. Intell. 47, 2388–2401. doi: 10.1109/TPAMI.2024.3524377 40030788

[B12] Ferentinos K. P. (2018). Deep learning models for plant disease detection and diagnosis. Comput. Electron. Agric. 145, 311–318. doi: 10.1016/j.compag.2018.01.00942574925

[B13] Fuentes A. Yoon S. Kim S. C. Park D. S. (2017). A robust deep-learning-based detector for real-time tomato plant diseases and pests recognition. Sensors 17, 2022. doi: 10.3390/s17092022 28869539 PMC5620500

[B14] Fujieda S. Takayama K. Hachisuka T. (2018). Wavelet convolutional neural networks. arXiv. doi: 10.48550/arXiv.1805.08620

[B15] Gu A. Dao T. (2023). Mamba: Linear-time sequence modeling with selective state spaces. arXiv. doi: 10.48550/arXiv.2312.00752

[B16] Han T. Dong Q. Wang X. Jia J. Sun L. Huang M. . (2026a). “ Learning depth guidance for camouflaged object detection without annotations”, in: ICASSP 2026–2026 IEEE international conference on acoustics, speech and signal processing (ICASSP) ( IEEE), 9957–9961. doi: 10.1109/ICASSP55912.2026.11462568

[B17] Han T. Dong Q. Wang X. Jia J. Wu G. Yang B. . (2026b). “ CamoQuery: Language-guided reasoning camouflaged object segmentation”, in: Proceedings of the 64th annual meeting of the association for computational linguistics (volume 1: Long papers), 22924–22941. doi: 10.18653/v1/2026.acl-long.1050

[B18] Han T. Wang X. Dong Q. Sun L. Huang M. Jia J. . (2026c). Dual-modal consistency learning for weakly supervised RGB-d camouflaged object detection with scribble annotations. Inf. Sci. 756, 123840. doi: 10.1016/j.ins.2026.12384042574925

[B19] Hu J. Shen L. Sun G. (2018). “ Squeeze-and-excitation networks”, in: Proceedings of the IEEE/CVF conference on computer vision and pattern recognition, Piscataway, NJ: IEEE, 7132–7141. doi: 10.48550/arXiv.1709.01507

[B20] Huang S. Hou Y. Liu L. Yu X. Shen X. (2025a). Real-time object detection meets DINOv3. arXiv. doi: 10.48550/arXiv.2509.20787

[B21] Huang S. Lu Z. Cun X. Yu Y. Zhou X. Shen X. (2025b). “ DEIM: DETR with improved matching for fast convergence”, in: Proceedings of the IEEE/CVF conference on computer vision and pattern recognition, 15162–15171. doi: 10.48550/arXiv.2412.04234

[B22] Huang Y. Zhao H. Wang J. (2024). YOLOv8-e: An improved YOLOv8 algorithm for eggplant disease detection. Appl. Sci. 14, 8403. doi: 10.3390/app1418840330654563

[B23] Huangfu Y. Huang Z. Yang X. Zhang Y. Li W. Shi J. . (2024). HHS-RT-DETR: A method for the detection of citrus greening disease. Agronomy 14, 2900. doi: 10.3390/agronomy1412290030654563

[B24] Huo Z. Yang X. Sun X. Zhang B. Liu D . (2026). EBGC-RTDETR: A lightweight model for eggplant disease detection with enhanced RT-DETR architecture. Smart Agric. Technol. 13, 101761. doi: 10.1016/j.atech.2025.10176142574925

[B25] Khanam R. Hussain M. (2024). Yolov11: An overview of the key architectural enhancements. arXiv. doi: 10.48550/arXiv.2410.17725

[B26] Khanramaki M. Askari Asli-Ardeh E. Kozegar E . (2021). Citrus pests classification using an ensemble of deep learning models. Comput. Electron. Agric. 186, 106192. doi: 10.1016/j.compag.2021.10619242574925

[B27] Kumar S. S. Raghavendra B. K. (2019). “ Diseases detection of various plant leaf using image processing techniques: A review”, in: 2019 5th international conference on advanced computing & communication systems (ICACCS) ( IEEE), 313–316. doi: 10.1109/ICACCS.2019.8728325

[B28] LeCun Y. Bengio Y. Hinton G. (2015). Deep learning. Nature 521, 436–444. doi: 10.1038/nature14539 26017442

[B29] Lei M. Li S. Wu Y. Hu H. Zhou Y. Zheng X. . (2025). YOLOv13: Real-time object detection with hypergraph-enhanced adaptive visual perception. arXiv. doi: 10.48550/arXiv.2506.17733

[B30] Li X. Sun W. Ji Y. Huang W. (2026). A plot-to-track association framework based on graph representation learning for compact HFSWR. IEEE Trans. Aerosp. Electron. Syst. 62, 12742–12760. doi: 10.1109/TAES.2026.370338525079929

[B31] Lin T.-Y. Goyal P. Girshick R. He K. Dollár P. (2020). Focal loss for dense object detection. IEEE Trans. Pattern Anal. Mach. Intell. 42, 318–327. doi: 10.1109/TPAMI.2018.2858826 30040631

[B32] Lin X. Peng J. Gan Z. Zhu J. Liu J. (2025). YOLO-master: MOE-accelerated with specialized transformers for enhanced real-time detection. arXiv. doi: 10.48550/arXiv.2512.23273

[B33] Liu L. Ouyang W. Wang X. Fieguth P. Chen J. Liu X. . (2020). Deep learning for generic object detection: A survey. Int. J. Comput. Vision 128, 261–318. doi: 10.1007/s11263-019-01247-430311153

[B34] Liu Y. Tian Y. Zhao Y. Yu H. Xie L. Wang Y. . (2024). VMamba: Visual state space model. Adv. Neural Inf. Process. Syst. 37, 103031–103063. doi: 10.48550/arXiv.2401.10166

[B35] Liu J. Wang X. (2025). EggplantDet: An efficient lightweight model for eggplant disease detection. Alexandria Eng. J. 115, 308–323. doi: 10.1016/j.aej.2024.12.03742574925

[B36] Liu Z. Wu D. Xu F. Du Z. Luo R. Li C. (2025). MSHLB-DETR: Transformer-based multi-scale citrus huanglongbing detection in orchards with aggregation enhancement. Horticulturae 11, 1225. doi: 10.3390/horticulturae1110122530654563

[B37] Liu H. Chen L. Lu X. Wang H. Bai L. Wang M. . (2026). A visual-textual mutual guidance fusion network for remote sensing visual question answering. Pattern Recognit. 176, 113258. doi: 10.1016/j.patcog.2026.11325842574925

[B38] Liu P. Zhang H. Zhang K. Lin L. Zuo W. (2018). “ Multi-level wavelet-CNN for image restoration”, in: Proceedings of the IEEE/CVF conference on computer vision and pattern recognition workshops, Piscataway, NJ: IEEE, 773–782. doi: 10.48550/arXiv.1805.07071

[B39] Mallat S. G. (1989). A Wavelet Tour of Signal Processing. 2nd ed. San Diego, CA: Academic Press.

[B40] Mallat S. (1999). A Wavelet Tour of Signal Processing (San Diego, CA: Elsevier). doi: 10.1016/B978-0-12-374370-1.X0001-8

[B41] Martinelli F. Scalenghe R. Davino S. Panno S. Scuderi G. Ruisi P. . (2015). Advanced methods of plant disease detection. A review. Agron. Sustain. Dev. 35, 1–25. doi: 10.1007/s13593-014-0246-130311153

[B42] Meshram A. T. Vanalkar A. V. Kalambe K. B. Badar A. M. (2022). Pesticide spraying robot for precision agriculture: A categorical literature review and future trends. J. Field Rob. 39, 153–171. doi: 10.1002/rob.2204342587375

[B43] Mohanty S. P. Hughes D. P. Salathé M. (2016). Using deep learning for image-based plant disease detection. Front. Plant Sci. 7, 1419. doi: 10.3389/fpls.2016.01419 27713752 PMC5032846

[B44] Nawaz M. Nazir T. Javed A. Amin S. T. Jeribi F. Tahir A. (2024). CoffeeNet: A deep learning approach for coffee plant leaves diseases recognition. Expert Syst. Appl. 237, 121481. doi: 10.1016/j.eswa.2023.12148142574925

[B45] Peng Y. Li H. Wu P. Zhang Y. Sun X. Wu F. (2025). “ D-FINE: Redefine regression task of DETRs as fine-grained distribution refinement”, in: in Proceedings of the Thirteenth International Conference on Learning Representations (ICLR 2025). Available online at: Available at: https://proceedings.iclr.cc/paper_files/paper/2025/hash/6cf58a87e3097e7d1f9be3e8693a93de-Abstract-Conference.html.

[B46] Qi J. Liu X. Liu K. Xu F. Guo H. Tian X. . (2022). An improved YOLOv5 model based on visual attention mechanism: Application to recognition of tomato virus disease. Comput. Electron. Agric. 194, 106780. doi: 10.1016/j.compag.2022.10678042574925

[B47] Redmon J. Divvala S. Girshick R. Farhadi A. (2016). “ You only look once: Unified, real-time object detection”, in: Proceedings of the IEEE conference on computer vision and pattern recognition, Piscataway, NJ: IEEE, 779–788. doi: 10.1109/CVPR.2016.91

[B48] Ren S. He K. Girshick R. Sun J. (2017). Faster r-CNN: Towards real-time object detection with region proposal networks. IEEE Trans. Pattern Anal. Mach. Intell. 39, 1137–1149. doi: 10.48550/arXiv.1506.01497 27295650

[B49] Safari M. Wang S. Wildman V. L. Hu M. Eidex Z. Chang C.-W. . (2026). Efficient vision mamba for MRI super-resolution via hybrid selective scanning. Med. Phys. 53, e70508. doi: 10.1002/mp.70508 42204783

[B50] Sapkota R. Cheppally R. H. Sharda A. Karkee M. (2025). YOLO26: Key architectural enhancements and performance benchmarking for real-time object detection. arXiv. doi: 10.48550/arXiv.2509.25164

[B51] Shi R. Wang Z. (2025). Research on the application of VMamba scanning algorithm in high-resolution remote sensing change detection. J. Comput. Commun. 13, 265–288. doi: 10.4236/jcc.2025.134017

[B52] Shi J. Zuo J. Zhou F. Gao L. Wang Q. Jiang A. (2018). Low-temperature conditioning enhances chilling tolerance and reduces damage in cold-stored eggplant (solanum melongena l.) fruit. Postharvest Biol. Technol. 141, 33–38. doi: 10.1016/j.postharvbio.2018.03.00742574925

[B53] Singh D. Jain N. Jain P. Kayal P. Kumawat S. Batra N. (2020). “ PlantDoc: A dataset for visual plant disease detection”, in: Proceedings of the 7th ACM IKDD CoDS and 25th COMAD, New York, NY: Association for Computing Machinery, 249–253. doi: 10.1145/3371158.3371196

[B54] Sun H. Fan W. Zhang J. Feng M. Wang F. Fu R. (2026). DFSNet: Directional feature aggregation and shape-aware supervision for eggplant pest and disease detection. Front. Plant Sci. 17, 1775987. doi: 10.3389/fpls.2026.1775987 41738045 PMC12926479

[B55] Sun H. Fu R. Kang D.-K. (2025a). A novel efficient eggplant disease detection method with multi-scale learning and edge feature enhancement. Front. Plant Sci. 16, 1666955. doi: 10.3389/fpls.2025.1666955 41049194 PMC12488578

[B56] Sun H. Fu R. Zhang Z. Sun H. Jeong Y. Lyu J. (2025b). Research on eggplant pest and disease detection in complex environments based on improved RT-DETR. BMC Plant Biol. 25, 1664. doi: 10.1186/s12870-025-07518-2 41315926 PMC12664156

[B57] Sun H. Wang R. Li Y. Yang L. Lin S. Cao X. . (2025c). “ SET: Spectral enhancement for tiny object detection,” in Proceedings of the 2025 IEEE/CVF Conference on Computer Vision and Pattern Recognition (CVPR), 4713–4723. doi: 10.1109/CVPR52734.2025.00444

[B58] Tian Y. Ye Q. Doermann D. (2026). YOLOv12: Attention-centric real-time object detectors. Adv. Neural Inf. Process. Syst. 38, 78433–78457. doi: 10.48550/arXiv.2502.12524

[B59] Vaswani A. Shazeer N. Parmar N. Uszkoreit J. Jones L. Gomez A. N. . (2017). “ Attention is all you need”, in: Advances in neural information processing systems, Red Hook, NY: Curran Associates, Inc. doi: 10.48550/arXiv.1706.03762

[B60] Wang A. Chen H. Liu L. Chen K. Lin Z. Han J. . (2024a). YOLOv10: Real-time end-to-end object detection. Adv. Neural Inf. Process. Syst. 37, 107984–108011. doi: 10.52202/079017-3429

[B61] Wang X. Girshick R. Gupta A. He K. (2018). “ Non-local neural networks”, in: Proceedings of the IEEE/CVF conference on computer vision and pattern recognition, Piscataway, NJ: IEEE, 7794–7803. doi: 10.48550/arXiv.1711.07971

[B62] Wang Z. Li C. Xu H. Zhu X. Li H. (2025b). “ Mamba-YOLO: A simple baseline for object detection with state space model”, in: Proceedings of the AAAI conference on artificial intelligence, Washington, DC: AAAI Press, 8205–8213. doi: 10.1609/aaai.v39i8.32885

[B63] Wang H. Sun S. Ren P. (2023). Meta underwater camera: A smart protocol for underwater image enhancement. ISPRS J. Photogramm. Remote Sens. 195, 462–481. doi: 10.1016/j.isprsjprs.2022.12.00742574925

[B64] Wang H. Zhang W. Ren P. (2024b). Self-organized underwater image enhancement. ISPRS J. Photogramm. Remote Sens. 215, 1–14. doi: 10.1016/j.isprsjprs.2024.06.01942574925

[B65] Wang H. Zhang W. Xu Y. Li H. Ren P. (2025a). WaterCycleDiffusion: Visual-textual fusion empowered underwater image enhancement. Inf. Fusion 127, 103693. doi: 10.1016/j.inffus.2025.10369342574925

[B66] Woo S. Park J. Lee J.-Y. Kweon I. S. (2018). “ CBAM: Convolutional block attention module”, in: Proceedings of the european conference on vision (ECCV), Cham: Springer, 3–19. doi: 10.48550/arXiv.1807.06521

[B67] Xie Z. Li C. Yang Z. Zhang Z. Jiang J. Guo H. (2024). YOLOv5s-BiPCNext, a lightweight model for detecting disease in eggplant leaves. Plants 13, 2303. doi: 10.3390/plants13162303 39204740 PMC11359194

[B68] Xu R. Yang S. Wang Y. Du B. Chen H. (2024). A survey on vision mamba: Models, applications and challenges. arXiv. arXiv:2404.18861. doi: 10.48550/arXiv.2404.18861

[B69] Yang Z. Guan Q. Yu Z. Xu X. Long H. Lian S. . (2025). MHAF-YOLO: Multi-branch heterogeneous auxiliary fusion YOLO for accurate object detection. arXiv. doi: 10.48550/arXiv.2502.04656

[B70] Yaseen M. (2024). What is YOLOv8: An in-depth exploration of the internal features of the next-generation object detector. arXiv. doi: 10.48550/arXiv.2408.15857

[B71] Yin D. Gontijo Lopes R. Shlens J. Cubuk E. D. Gilmer J. (2019). A fourier perspective on model robustness in computer vision. Adv. Neural Inf. Process. Syst. 32, 13255–13265. doi: 10.48550/arXiv.1906.08988

[B72] Zhao Y. Lv W. Xu S. Wei J. Wang G. Dang Q. . (2024). “ DETRs beat YOLOs on real-time object detection”, in: Proceedings of the IEEE/CVF conference on computer vision and pattern recognition, Piscataway, NJ: IEEE, 16965–16974. doi: 10.1109/CVPR52733.2024.01605

[B73] Zhu X. Su W. Lu L. Li B. Wang X. Dai J. (2020). Deformable DETR: Deformable transformers for end-to-end object detection. arXiv. doi: 10.48550/arXiv.2010.04159

